# Inflammatory mediators in intra-abdominal sepsis or injury – a scoping review

**DOI:** 10.1186/s13054-015-1093-4

**Published:** 2015-10-27

**Authors:** Zhengwen Xiao, Crystal Wilson, Helen Lee Robertson, Derek J. Roberts, Chad G. Ball, Craig N. Jenne, Andrew W. Kirkpatrick

**Affiliations:** Regional Trauma Services, Foothills Medical Centre, 1403 – 29 Street NW, T2N 2T9 Calgary, AB Canada; Health Sciences Library, Health Sciences Centre, University of Calgary, 3330 Hospital Drive NW, T2N 4N1 Calgary, AB Canada; Department of Surgery, Foothills Medical Centre, University of Calgary, 1403 – 29 Street NW, T2N 2T9 Calgary, AB Canada; Department of Community Health Sciences (Division of Epidemiology), University of Calgary, 3280 Hospital Drive Northwest, T2N 4Z6 Calgary, AB Canada; Department of Critical Care Medicine, Foothills Medical Centre, University of Calgary, 3134 Hospital Drive NW, T2N 5A1 Calgary, AB Canada; Department of Microbiology, Immunology and Infectious Diseases, University of Calgary, 3280 Hospital Drive NW, T2N 4N1 Calgary, AB Canada

## Abstract

**Introduction:**

Inflammatory and protein mediators (cytokine, chemokine, acute phase proteins) play an important, but still not completely understood, role in the morbidity and mortality of intra-abdominal sepsis/injury. We therefore systematically reviewed preclinical and clinical studies of mediators in intra-abdominal sepsis/injury in order to evaluate their ability to: (1) function as diagnostic/prognostic biomarkers; (2) serve as therapeutic targets; and (3) illuminate the pathogenesis mechanisms of sepsis or injury-related organ dysfunction.

**Methods:**

We searched MEDLINE, PubMed, EMBASE and the Cochrane Library. Two investigators independently reviewed all identified abstracts and selected articles for full-text review. We included original studies assessing mediators in intra-abdominal sepsis/injury.

**Results:**

Among 2437 citations, we selected 182 studies in the scoping review, including 79 preclinical and 103 clinical studies. Serum procalcitonin and C-reactive protein appear to be useful to rule out infection or monitor therapy; however, the diagnostic and prognostic value of mediators for complications/outcomes of sepsis or injury remains to be established. Peritoneal mediator levels are substantially higher than systemic levels after intra-abdominal infection/trauma. Common limitations of current studies included small sample sizes and lack of uniformity in study design and outcome measures. To date, targeted therapies against mediators remain experimental.

**Conclusions:**

Whereas preclinical data suggests mediators play a critical role in intra-abdominal sepsis or injury, there is no consensus on the clinical use of mediators in diagnosing or managing intra-abdominal sepsis or injury. Measurement of peritoneal mediators should be further investigated as a more sensitive determinant of intra-abdominal inflammatory response. High-quality clinical trials are needed to better understand the role of inflammatory mediators.

**Electronic supplementary material:**

The online version of this article (doi:10.1186/s13054-015-1093-4) contains supplementary material, which is available to authorized users.

## Introduction

Sepsis is a syndrome that results from the interactions between the host and insults (pathogen, injury) leading to the production/release of biochemical mediators and the triggering of inflammatory cascades [1]. Tissue damage and shock lead to the extracellular release of damage-associated molecular patterns (DAMPs), which evoke a systemic inflammatory response syndrome (SIRS) and hypoxia, reduce resistance to infection, and increase risk of sepsis [2–4]. Moreover, microbial infection produces pathogen-associated molecular patterns (PAMPs) that drive septic inflammation. DAMPs and PAMPs share a number of conserved families of pattern recognition receptors (PRRs) including the prototypical PRR family, the Toll-like receptors (TLRs). Activation of TLRs on immune cells and endothelial cells leads to the release of pro- and anti-inflammatory mediators, which trigger excessive inflammation, sepsis and multiple organ failure (MOF) [5, 6].

Intra-abdominal infection and injury are common causes of sepsis in surgical intensive care unit (ICU) patients, with high rates of mortality [7–9]. Early diagnosis and treatment of infection in this group of patients is associated with improved outcome and reduced mortality. However, the clinical definitions of sepsis are nonspecific and often result in delay in diagnosis and therapy. One of the most challenging obstacles to the treatment of intra-abdominal infection and injury has been establishing the diagnosis and differentiating sepsis with bacterial infection from “sterile” SIRS [10].

Given that both intra-abdominal infection and injury can incite hyperinflammatory status and sepsis, and the inflammatory response to injury is often clinically indistinguishable from sepsis, many studies have sought to identify biomarkers or mediators to aid in the diagnosis and management of sepsis [11, 12]. The purpose of this scoping review was therefore to systematically review preclinical and clinical studies of inflammatory mediators in abdominal sepsis and injury in order to evaluate their ability to: (1) provide earlier diagnosis of SIRS, sepsis or severe sepsis, or predict complications or outcomes; (2) serve as therapeutic targets for randomized controlled trials (RCTs); (3) illuminate the mechanisms of pathogenesis of sepsis or injury-related organ dysfunction.

## Methods

We developed a protocol according to the Arksey and O’Malley methodological framework for conducting scoping reviews with modifications [13]. Scoping reviews entail the systematic selection, collection and summarization of existing knowledge in a broad thematic area for the purpose of identifying if there is sufficient evidence to conduct a full synthesis or further research is needed. Contrary to systematic reviews, in scoping reviews authors do not typically assess the quality of included studies or perform a meta-analysis [13].

### Search strategy

Two investigators (ZX, AWK) created a preliminary search strategy that was subsequently refined by a medical librarian with extensive systematic review experience (HLR). Relevant articles were identified by searching the following databases from the first date available until August 2014: Ovid MEDLINE; Ovid EMBASE; PubMed; Cochrane Database of Systematic Reviews, and Cochrane Central Register of Controlled Trials. Combinations of the following search terms were used (see Table S1 in Additional file 1 for detailed search strategies): abdominal injuries, intraabdominal inflammation, intraabdominal sepsis, intraabdominal infections, intraabdominal hypertension, peritonitis, sepsis, septic shock, systemic inflammatory response syndrome, bacteremia, multiple organ failure, inflammation mediator, cytokine, interleukin, and biological markers. Appropriate wildcards were used in all searches to account for plural words and variations in spelling. Additional articles were identified by manually searching the bibliographies of those articles identified in the searches until December 2014.

### Article selection

Two investigators (ZX, CW) independently screened the titles and abstracts of all identified citations. We included all articles that were original studies (controlled trials, cohort study, case series, case-control) assessing inflammatory or protein mediators in intra-abdominal sepsis or injury. Participants included patients (10 or more) admitted for surgery with peritonitis or abdominal injury, or animal models of abdominal sepsis/injury. Participants required measurement of mediator(s) from blood and/or peritoneal fluid for inclusion. Articles were excluded if not reporting original data, such as reviews, letters, and conference abstracts (no full text available). Disagreements between the two assessors were resolved by consensus.

### Article review, data charting and reporting

The full texts of the remaining articles were independently reviewed by the same two authors. The two investigators extracted data independently using a predesigned electronic data extraction form. Assessors were not blinded to author or journal name [14]. We extracted the following data from included studies: (1) study design and setting; (2) study participant characteristics, including age, primary patient diagnosis (for example, trauma, intra-abdominal sepsis, or source of infection), and severity of illness (for example, acute physiology and chronic health evaluation II (APACHE II) [15], sequential organ failure assessment (SOFA) [16], and injury severity score (ISS) [17]; (3) severity of sepsis [10]; (4) the number of participants, and intervention groups; (5) details of animal models; (6) details of reported mediators; and (7) the outcomes.

Results were first mapped based on their characteristics, such as study design, sample size, preclinical or clinical studies. Owing to the significant variability in study design, sample size, analytical technique and outcome measures, it was impossible to perform a full synthesis analysis of the findings. Therefore, studies were presented in tables based on thematic characteristics of the articles. In addition, the level of evidence for a mediator as a biomarker was discussed based on the “best-evidence synthesis” guideline used in review studies (see Table S2 in Additional file 2) [18].

### Risk of bias assessment

Risk of bias of clinical controlled studies was assessed using the guidelines proposed by the Cochrane Collaboration Back Review Group [19, 20]. These guidelines assist in evaluating study patient participation and treatment allocation, outcome, and confounding factor measurement.

## Results

**Fig. 1:**
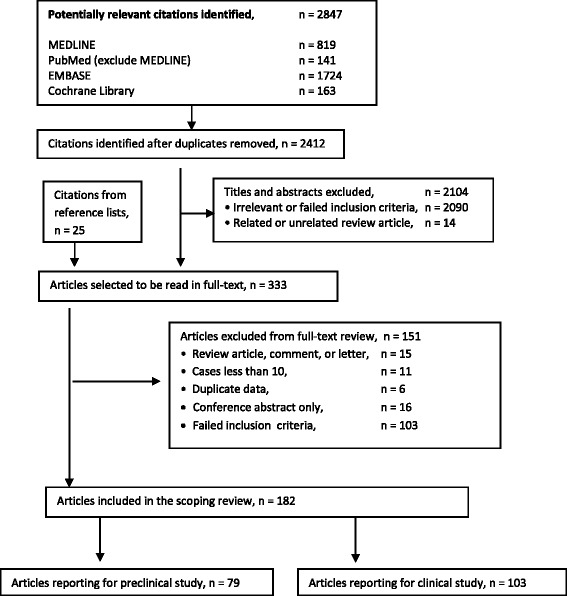
Flow chart of steps for study selection

The literature search identified 2412 studies (after duplicates removed) and the search from reference lists identified an additional 25 articles. The review of abstracts led to retrieval of 333 full-text articles for assessment. We selected 182 articles for inclusion of this review (Fig. 1).

### Characteristics of the articles

Table 1 summarizes the characteristics of the articles on mediators in intra-abdominal sepsis or injury. Most articles were published in the last two decades (92 %). There were 103 clinical studies (57 %) and 79 preclinical studies (43 %). Among the clinical studies, 63 articles reported mediators as biomarkers for diagnosis on or predicting outcomes of intra-abdominal sepsis; 23 articles described the kinetic change of mediators in intra-abdominal sepsis or injury; and eight were mechanistic studies. Only nine intervention studies with moderate methodological quality were identified concerning intra-abdominal sepsis management. Of the 79 preclinical studies, rodent models and porcine models were the most common animal models used.

**Table 1:** Characteristics of articles on inflammatory/protein mediators in intra-abdominal sepsis or injury

Characteristics	Number (%) of 182 articles
Type of article	
Clinical study	103 (57 %)
Biomarker or diagnosis study	63 (35 %)
Intervention study	9 (5 %)
Mechanistic or kinetic study	31 (17 %)
Study design	
Randomized controlled trials	4
Nonrandomized controlled trials	5
Cohort study	36
Case-control study	29
Case series	29
Abdominal sepsis	64
Abdominal injury or ischemia-reperfusion	27
Mixed sepsis and injury	12
Preclinical study	79 (43 %)
Intervention study	32 (17 %)
Mechanistic study	47 (26 %)
Study design	
Rodent model	69
Porcine model	7
Canine model	1
Rabbit model	1
Baboon model	1
Injury or ischemia-reperfusion	26
Abdominal sepsis	44
Mixed injury and sepsis	9
Year of publication	
1985–1994	14 (8 %)
1995–2004	65 (35 %)
2005–2014	103 (57 %)
Country of origin	
European countries	80 (44 %)
United States of America	44 (24 %
Other countries	58 (32 %)

### Description of the studies and mediators

Table S3 in Additional file 3 summarizes the clinical studies of mediators as biomarkers in diagnosing infection or predicting outcomes of intra-abdominal sepsis and injuries. Studies of perioperative kinetic changes of mediators are also included in this table. In general, before 1992, C-reactive protein (CRP) was the most common mediator studied [21–26]. Thereafter, interleukins (IL-6, -8, -10) and tumor necrosis factor alpha (TNF-α) were on the top list of the reported mediators [27–106]. Since 2000, procalcitonin (PCT) was often examined as a clinically promising biomarker [49, 57, 64, 66, 74, 77, 78, 88, 93, 95, 100, 101, 106]. Notably PCT was the only biomarker being explored through randomized-type designs. More recently, DAMPs and endothelial dysfunction molecules had been added to the ever-growing list of mediators [3, 63, 80, 82, 84, 85, 91, 99, 105].

### C-reactive protein

There have been 33 studies exploring CRP as a marker for abdominal infection or complications after surgery [21, 22, 24–27, 29, 32, 34, 39, 47, 49, 51–53, 57, 61, 64, 66, 68, 70, 79, 86–88, 90, 92, 95, 96, 98, 100, 103, 106]. Kinetics studies demonstrated that serum CRP levels begun to elevate on postoperative day 1 (POD1), peaked from POD2 to POD3 (approximately 12–24 hours after peak levels of IL-6), and then declined to baseline levels on POD5 providing there was no complication or infection after major abdominal surgery [29, 39, 52, 53, 57, 79, 90, 96]. Four reports suggested that persistent levels of more than 100 mg/l after POD5 indicate abscess formation or other septic complications (e.g., anastomotic leakage) [21, 92, 98, 103]. Mustard et al*.* reported an accuracy of 75 % for detecting infection when CRP was greater than 15 mg/l after POD4 [24]. However, other studies reported a limited role for CRP to diagnose sepsis or predict outcomes in other clinical scenarios [34, 49, 51, 64, 87, 88, 96].

### Procalcitonin

Twelve trials (including two randomized controlled studies) explored PCT as an indicator to diagnose infection, predict outcomes, or guide treatment of abdominal sepsis or injury [49, 57, 64, 66, 74, 77, 78, 88, 93, 95, 100, 101]. Serum PCT increased immediately after surgical injury, peaked on POD1, and declined to half its peak level from POD2 to POD3 after uncomplicated major abdominal surgery [57, 66, 77, 100]. Reith et al*.* performed a large case-control study of 246 patients with abdominal sepsis [49]. The results from this study suggested that serum PCT appeared to be a good predictor of severity and mortality. A reduction of serum PCT on POD1 to POD4 correlated with improvement in sepsis [49]. Similar findings were reported by Mokart et al*.* leading to the suggestion that a cutoff value of 1.1 ng/ml serum PCT on POD1 was a good predictor of subsequent sepsis in patients undergoing major surgery [64]. Four studies suggested that PCT clearance kinetics appear to be a better indicator than a single cutoff value to diagnose septic complications or predict outcomes. Persistently high PCT in plasma was associated with infection or with a significant increase in mortality in patients with sepsis in those studies [77, 88, 93, 95]. Therefore, PCT had been used as a guide for imaging or interventions, or antibiotic therapy for patients with abdominal sepsis [74, 78]. Importantly however, other studies have not consistently confirmed PCT as an accurate marker for sepsis or to predict patient’s response to the initial treatment [100, 101].

### Interleukin-6

Fifty-five of 86 studies examined IL-6, alone, or in conjunction with other cytokines (TNF-α, IL-1, -2, -4, -8, -10, -12, -18). IL-6 levels in plasma are rapidly dynamic and peaking at maximum from wound closure to POD1. IL-6 generally declined on POD1, and was back to baseline on POD3 [29, 39, 40, 42, 43, 45, 49, 52–54, 61, 62, 75, 79, 90]. Patients with infection showed higher serum IL-6 than those without complication after surgical injury. However, the role of IL-6 as a marker to diagnose sepsis or predict outcomes remains uncertain, and a wide range of cutoff values had been used (from 12 to 2760 pg/ml). Fifteen studies reported that IL-6 appeared to be an indicator for sepsis or for predicting outcome/mortality [27, 36, 46–48, 50, 60, 62, 64, 70, 81, 82, 87, 89, 91]. Conversely however, nine studies suggested IL-6 was a poor marker of disease severity [28, 35, 55, 65, 66, 72, 80, 83, 96]. The role of other cytokines as markers, such as IL-1β, -2, -4, -8, -10, -12, -18, and TNF-α also remains elusive.

### DAMPs

Recent studies have reported that DAMPs can act as proinflammatory mediators. Cohen et al*.* demonstrated high mobility group box protein 1 (HMGB1) was elevated in plasma early after injury and shock. Levels of HMGB1 correlated with injury severity and morbidity in trauma patients [3]. Similar results had been reported by Manganelli et al*.* in surgical patients [84]. HMGB1 level of the patients with peritonitis was higher than healthy controls [85, 99]. Mitochondrial DNA (mtDNA) has been proposed to be an inflammatory mediator, which was elevated early after trauma [105].

### Intraperitoneal mediators

All studies that examined both systemic (blood) and peritoneal levels of mediators demonstrated that peritoneal mediator levels were 10–1000 times higher than systemic values in patients with peritonitis [33, 38, 51, 54, 61, 65, 67, 69, 71, 76, 89, 102]. This suggests that measurement of peritoneal cytokines may be another and potentially more important method to determine and follow the patient’s inflammatory reaction.

### Mechanistic studies

Table S4 in Additional file 4 summarizes the eight clinical mechanistic studies that have been performed [107–114]. Moore et al. reported endotoxin could not be detected in portal or systemic blood in the first 48 hours post injury, and no differences were reported in terms of portal and systemic blood levels of IL-6 and TNF-α between patients who developed MOF or not [107]. However, Adembri et al*.* carried out a small case-control study, and indicated a significant increase in blood IL-6 after reperfusion in aortic abdominal aneurysm surgery patients, which was followed by a significant reduction of lung function [111]. Sperry et al*.* reported blood IL-6 levels were statistically higher in males than females after injury [113]. In patients with severe peritonitis, the percentage of peritoneal neutrophils that engulfed *Escherichia coli* bacteria was significantly depressed when compared to patients without septic shock [108]. Moreover, severe peritonitis provoked an early pulmonary expression of chemoattractants, which enhanced neutrophil sequestration and activation in the lung [114].

### Intervention studies

Table S5 in Additional file 5 lists the nine clinical intervention studies [115–123]. Four of which were randomized controlled trials (RCT). Three of the RCTs were from a single hospital comparing open surgery versus laparoscopy for treating perforated appendicitis, perforated peptic ulcer, or cholecystitis with sepsis [120–122]. Bakker et al*.* compared surgical to endoscopic necrosectomy for the treatment of infected necrotizing pancreatitis [119]. From these studies, the authors concluded that open surgery after peritonitis increased the incidence of bacteremia, endotoxemia, and systemic inflammation compared with laparoscopy. However, despite the direct linkage between endotoxin and inflammation, the use of polymyxin B-immobilized fiber to remove endotoxin or inflammatory mediators by direct hemoperfusion has proven to be of limited clinical value [116].

Kirkpatrick et al*.* performed a RCT looking at mediator levels in 45 patients with intra-abdominal sepsis or injury who needed open abdomen management, to determine whether active negative pressure peritoneal therapy with the ABThera device reduces systemic or peritoneal inflammation [123]. In this study, a significant 90-day mortality difference (*P* = 0.04) was observed among patients randomized to the ABThera (21.7 %) versus Barker’s vacuum pack (50 %). Interestingly, there was no significant difference in clearance of the peritoneal or plasma IL-6 (or a number of other cytokines) between 24 and 48 hours among patients randomized to the ABThera versus Barker’s vacuum pack.

### Animal studies

Table S6 in Additional file 6 summarizes the mechanistic studies of mediators in animal models [124–170]. Thirty-nine of the 47 studies used rodents to develop abdominal sepsis models (i.e., cecum ligation and puncture (CLP)) [124, 125, 127, 128, 131–133, 136–138, 143, 145, 151, 154, 156, 157, 159, 162, 164, 165, 167–170], or shock-resuscitation, or abdominal compartment syndrome (ACS), or two-hit models [126, 130, 132, 135–140, 142, 144, 146–150, 152, 160–162, 166]. The remaining utilized swine [129, 134, 155, 158, 163], baboons [141], or dogs [153]. These studies sought to address a wide range of mechanisms using various study designs. Four studies reported that balance of inflammatory mediators was closely related to severity and outcome of sepsis, and in these models IL-10 played a critical role to attenuate local and systemic mediators in animals with abdominal sepsis or injury [125–127, 140]. Ten reports used two-hit models, including shock (or ischemia) plus CLP or ACS [132, 134, 136, 137, 139, 155, 158, 163]. Overall these studies indicated that trauma or shock (the first hit) initiated an early proinflammatory response, resulting in priming and activation of neutrophils in peritoneum or other organs (lung, liver, kidney). The second insult (infection or ACS) further exaggerated this response, leading to MOF and mortality.

Studies also demonstrated that local (peritoneal) inflammatory mediator levels were not accurately reflected by systemic (blood) mediator levels. Generally, peritoneal mediator levels were much higher than the systemic levels, and thus might be a truer or better indicator of the local inflammatory response and outcomes [131, 154, 158, 163]. The release of local mediators into the systemic circulation may help precipitate the deleterious effects of SIRS and sepsis. The lymphatic pathway appears to be a limiting step to systemic inflammatory response and subsequent lung injury following visceral ischemia and reperfusion [141, 153, 160].

Knockout animal studies demonstrated IL-10 was a potent anti-inflammatory mediator, resulting in the attenuation of lung neutrophil infiltration and injury after visceral ischemia [140]. ICAM-1-deficient mice undergoing CLP peritonitis showed better survival with less tissue damage and lower levels of plasma cytokines than their wild-type littermates [143]. Deficiency for the chemoattractant MIP-1α attenuated Kupffer cell cytokine production and systemic mediators following hemorrhage shock and resuscitation [148].

Table S7 in Additional file 7 lists the preclinical intervention studies performed on intra-abdominal sepsis models [171–202]. Use of anti-TNF antibody in mice subjected to CLP sepsis significantly reduced TNF-α bioactivity but interestingly did not reduce mortality [171, 172]. Administration of androgen receptor blocker and estrogen receptor agonists (flutamide, dehydroepiandrosterone) ameliorated a male gender-related immunity imbalance after hemorrhagic shock, reduced cytokine production resulting in improved animal survival during a subsequent septic challenge [144, 147, 173, 174, 179, 185]. Treatment with anti-HMGB1 antibody in mice after shock-resuscitation improved survival and ameliorated gut barrier dysfunction with lower blood cytokines [186]. Intravenous administration of a ω-3 fatty acid improved survival in mice CLP sepsis, and reduced blood cytokine levels [196]. Kubiak et al*.* reported that systemic and peritoneal inflammation was reduced with less organ damage in pigs with peritonitis treated by negative pressure therapy [190]. Although animal studies appear promising, one should keep in mind that many of these studies are small in nature. Furthermore, many limitations remain in translation of animal data to clinical application. In animal models, the insults are controlled; interventions can begin shortly after infection. In humans, the diagnosis is usually made much later. Thus it is essential that more evidence for the use of these interventions is gathered using clinical feasible models and larger sample size studies.

## Discussion

This scoping review focused on examining the reported role of inflammatory and protein mediators in intra-abdominal sepsis or injuries. In surgical ICU patients, traumatic injuries and septic conditions can be simultaneously or subsequently present. Each can be associated with profound and dynamic production of bioactive mediators that are at the present poorly understood, particularly in terms of their kinetics and overall interaction with the host inflammatory response. This inflammatory response is a complex and multifaceted process. Traumatic injuries induce overwhelming reactions in the immunological and neurohormonal systems. Innate immunocytes are activated by hypoxia stress and endogenous signals (DAMPs) released by damaged tissues [4, 6, 203]. These reactions are thought to represent attempts to adjust physiology for maintenance of homeostasis. These responses however often result in positive feedback loops leading to excessive cytokine production and uncontrolled inflammation. In the early phase following injury, the response is regulated by acute phase reactants, proinflammatory mediators (TNF-α, IL-1, -6, -8, -18), and the activation of endothelial cells (expression of P- and E-selectins, ICAM-1, VCAM-1), leading to a so-called “sterile” systemic inflammatory response or SIRS. At the same time, anti-inflammatory mediators (IL-10) are released in an attempt to balance the proinflammatory reaction. Polytrauma patients with excessive SIRS can not only progress to multiple organ damage and failure (MOF), but also develop persistent inflammation, immunosuppression, and catabolism syndrome (PICS), which lead to infectious sepsis resulting in release of additional DAMPs and PAMPs, perpetuating a vicious cycle of inflammation [4, 203]. Therefore, during this late phase post injury, trauma patients are prone to develop infective complications with high mortality rate. Injuries to the abdomen and visceral organs are common clinical scenarios, and are usually complicated with intra-abdominal sepsis [123]. The objective of this scoping review was to search for current evidence of mediators as biomarkers and their roles in intra-abdominal sepsis or injury.

Although animal studies suggest that mediators play a critical role in both the pathogenesis and potential management in intra-abdominal sepsis/injury, the clinical evidence to support measuring mediators as biomarkers to discriminate SIRS from sepsis is conflicting.

Due to the heterogeneity of included studies, we discussed the overall results based on the “best-evidence synthesis” rating, which is widely used in narrative and systematic reviews [18]. Moderate evidence supports utilizing serum CRP as a biomarker for diagnosing acute appendicitis with sepsis [25, 26, 32, 47, 70]; or as a potential indicator for predicting complications (abscess, anastomotic leaks) after major abdominal surgery [21, 92, 98, 103]. A normal CRP response to therapy, or absence of secondary rise after surgery, may help to exclude infection [24]. It is important to note that no standard cutoff value was available, and some studies have reported negative results for using CRP as a biomarker [64, 86, 96].

Furthermore, although the serum PCT value appears to predict severity of septic complications [49, 64], it does not appear to be an ideal biomarker to discriminate SIRS from sepsis. Persistently high PCT levels appear to be associated with a significant increase in mortality in patients with sepsis [77, 88, 93, 95]. The role of using serum PCT as a guide to monitor patients’ response to therapy remains to be determined [74, 78].

Sepsis further increased IL-6 levels over the already elevated levels from the original injury. Although moderate level of evidence supports IL-6 to be a useful indicator for sepsis or severity [48, 64, 87, 91], it is important to note that almost half of the studies for this cytokine do not support the use of IL-6 as a good sepsis biomarker. Currently, no evidence is available to support the use of TNF-α or other cytokines in diagnosing sepsis or predicting outcomes. Recent studies have reported endogenous DAMPs (mtDNA, HMGB1) released as a consequence of tissue injury or infection appear to be promising biomarkers [3, 84, 85, 99, 105]; however, the evidence supporting their role is still limited.

It appeared to be futile to specifically target a single inflammatory or protein mediator in an effort to mitigate disease and improve outcomes. It is more likely that identifying promising biomarkers will only be possible when a number of mediators are analyzed as a profile [204, 205].

It is currently unknown if the peritoneal cavity functions to restrain the inflammatory mediators or to act as a reservoir of mediators. Evidence has shown that peritoneal fluid functions as a priming and activating stimulus for neutrophils both in the peritoneum and in remote organs (lung, liver, kidneys) after injury, enhancing the incidence of MOF upon subsequent infection [136, 163]. Moreover, early removal of peritoneal fluid appeared to reduce systemic inflammation and organ dysfunction [123, 141, 153, 160, 190]. This notion supports the recent guidelines for management of intra-abdominal infections proposed by the World Society of Emergency Surgery [206].

Finally, based on the evidence from animal models that injury followed by a second insult (infection, or ACS) substantially enhanced the inflammatory response and organ damage [134, 136, 158, 163, 190], it is essential to avoid or timely intervene upon secondary insults for trauma patients to improve outcomes.

This review has limitations. First, despite the search of multiple databases using comprehensive search strategies with the assistance of a medical librarian, our search has excluded studies assessing extra-abdominal sepsis or injury. Moreover, limited abdominal trauma studies were available; the majority of which focused on surgical injuries. Importantly, the exclusion of studies assessing other kinds of sepsis can present a selection bias. Second, due to the inconsistencies in study design, it proved difficult to extract accurate data from all studies, even with the assistance of predefined data abstraction tools. Furthermore, only a small number of the included studies actually examined biomarkers for both sensitivity and specificity.

## Conclusions

Systemic inflammatory response syndrome is the expected immunologic response to both infection and traumatic injuries, which makes the distinction between infectious sepsis and sterile SIRS exceedingly difficult. Although animal studies suggest that mediators play an important role, the clinical use of mediators as biomarkers in discriminating SIRS from sepsis in intra-abdominal injury or sepsis is conflicting. Persistent elevation of CRP or PCT levels in blood appears to be correlated with septic complications. The role of IL-6 as a biomarker in intra-abdominal sepsis is controversial. Limited evidence suggests endogenous molecules may be candidate biomarkers of sepsis or injury. Importantly, peritoneal mediator levels cannot be proportionately reflected by the systemic levels. Victims subjected to trauma followed by infection show more excessive inflammatory response and organ damage than those to trauma only. To date, therapies targeting a specific inflammatory or protein mediator in abdominal sepsis have shown poor results. Interventions aiming to remove peritoneal fluid/mediators and improve outcomes are still investigational. Large sample size randomized controlled trails are desirable to identify the “ideal” biomarker(s) and understand the roles of mediators in intra-abdominal sepsis and injury.

## Key messages

- Preclinical studies demonstrate that intra-abdominal injury/ischemia, especially with subsequent infection, induces an excessive inflammatory/protein mediator production and uncontrolled inflammation in the peritoneum and remote organs.
- Both preclinical and clinical studies have shown the peritoneal mediator levels are much higher than the blood levels, and the release of these mediators (mostly via the lymphatic pathway) into the systemic circulation may precipitate the deleterious effects of sepsis and multiple organ dysfunction.
- Normal postoperative clearance kinetics of CRP, PCT, or IL-6 may help rule out infection or complications.
- The clinical role of measuring inflammatory/protein mediators as biomarkers to diagnose abdominal infection or predict outcomes of abdominal sepsis or injury remains to be established. High-quality clinical trials are warranted to determine the role of mediators in intra-abdominal sepsis and injury.

## Additional files

Additional file 1: Table S1. Search strategies. (DOCX 17 kb) Additional file 2: Table S2. Best-evidence synthesis. (DOCX 15 kb) Additional file 3: Table S3. Clinical biomarkers. (DOCX 76 kb) Additional file 4: Table S4. Clinical mechanism. (DOCX 19 kb) Additional file 5: Table S5. Clinical intervention. (DOCX 22 kb) Additional file 6: Table S6. Preclinical mechanism. (DOCX 46 kb) Additional file 7: Table S7. Preclinical intervention. (DOCX 36 kb)

## Abbreviations

ACS: abdominal compartment syndrome
CLP: cecum ligation and puncture
CRP: C-reactive protein
DAMPs: damage-associated molecular patterns
HMGB1: high mobility group box protein 1
ICU: intensive care unit
IL: interleukin
mtDNA: mitochondrial DNA
MOF: multiple organ failure
PAMPs: pathogen-associated molecular patterns
PCT: procalcitonin
PICS: persistent inflammation, immunosuppression, and catabolism syndrome
POD: postoperative day
PRRs: pattern recognition receptors
RCT: randomized controlled trial
SIRS: systemic inflammatory response syndrome
TLRs: Toll-like receptors
TNF: tumor necrosis factor
