# Supplementary material for: Inflammatory mediators in intra-abdominal sepsis or injury – a scoping review
Source: Crit Care. 2015 Oct 27;19:373. doi: 10.1186/s13054-015-1093-4 (PMC4623902; doi:10.1186/s13054-015-1093-4)
Supplement: Additional file 1: Table S1. — Search strategies. (DOCX 17 kb) [file 13054_2015_1093_MOESM1_ESM.docx]

**Table S1. Details of electronic database search strategies**

| **Ovid MEDLINE and MEDLINE In-Process & Other Non-indexed Citations, Ovid EMBASE, and the Cochrane Database of Systematic Reviews** | |
| --- | --- |
| 1 intraabdominal inflammation.mp.  2 intra-abdominal inflammation.mp.  3 intraabdominal sepsis.mp.  4 intra-abdominal sepsis.mp.  5 exp Intraabdominal Infections  6 intraabdominal infection*.mp.  7 intra-abdominal infection*.mp.  8 exp Intra-abdominal hypertension  9 intra-abdominal hypertension*.mp.  10 intraabdominal hypertension*.mp.  11 abdominal compartment syndrome*.mp.  12 exp Peritonitis  13 peritonitis.mp.  14 exp Abdominal Injuries  15 abdominal injur*.mp.  16 intraabdominal injur*.mp.  17 intra-abdominal injur*.mp.  18 or/1-17  19 exp Shock, Septic  20 septic shock.mp.  21 exp Sepsis  22 sepsis.mp.  23 septicemia.mp.  24 exp Infection  25 infection*.mp.  26 exp Inflammation  27 inflammation.mp.  28 exp Bacteremia  29 bacteremia*.mp.  30 exp Sepsis syndrome  31 sepsis syndrome*.mp.  32 or/19-31 (1873620)  33 intraabdominal.mp.  34 intra-abdominal.mp.  35 abdominal.mp.  36 exp Abdomen  37 exp Peritoneal Cavity  38 peritoneal.mp.  39 or/33-38  40 32 and 39  41 exp Systemic inflammatory response syndrome  42 systemic inflammatory response*.mp. | 43 exp Multiple organ failure  44 multiple organ failure.mp.  45 MODS.mp.  46 multiple organ dysfunction syndrome*.mp.  47 or/41-46  48 18 or 40 or 47  49 exp Inflammation Mediators  50 inflammation mediator*.mp.  51 procalcitonin.mp.  52 exp C-Reactive Protein  53 c-reactive protein.mp.  54 exp Haptoglobins  55 haptoglobin*.mp.  56 haptoglobulin*.mp.  57 exp Tissue Plasminogen Activator  58 tissue plasminogen activator*.mp.  59 t-plasminogen activator*.mp.  60 cytokine*.mp.  61 interleukin*.mp.  62 interleukins/ or interleukin-1/ or interleukin-6/ or interleukin-8/ or interleukin-10/  or exp interleukin-12/ or interleukin-15/ or interleukin-17/ or exp interleukin-23/  63 exp Tumor Necrosis Factor-alpha/  64 tumor necrosis factor.mp.  65 Tumour Necrosis Factor-alpha.mp.  66 Tumour Necrosis Factor.mp.  67 exp Biological Markers/  68 biological marker*.mp.  69 biomarker*.mp.  70 biologic marker*.mp.  71 or/49-69  72 cytokine concentration*.mp.  73 exp Abdomen/su [Surgery]  74 exp Abdominal Cavity/su [Surgery]  75 exp Abdominal Wall/su [Surgery]  76 ((abdominal or abdomen) adj3 surger*).mp.  77 (intraabdominal adj3 surger*).mp.  78 (intra-abdominal adj3 surger*).mp.  79 exp Laparotomy/  80 or/72-79  81 48 and 71 and 80 |

‘mp’ denotes title, abstract, name of substance word, subject heading word, unique identifier; ‘exp’ denotes that the search will be exploded; ‘*’ indicates that a wild card search will be performed.
