# Supplementary material for: Inflammatory mediators in intra-abdominal sepsis or injury – a scoping review
Source: Crit Care. 2015 Oct 27;19:373. doi: 10.1186/s13054-015-1093-4 (PMC4623902; doi:10.1186/s13054-015-1093-4)
Supplement: Additional file 2: Table S2. — Best-evidence synthesis. (DOCX 15 kb) [file 13054_2015_1093_MOESM2_ESM.docx]

**Table S2. Criteria for determining the level of evidence for best evidence synthesis (BES).**

| Level of evidence | Criteria for inclusion in BES |
| --- | --- |
| Strong evidence | Generally consistent findings in:   1. 1 high quality randomized controlled study 2. >2 high quality cohort studies |
| Moderate evidence | Generally consistent findings in:   1. 1 cohort study   and >2 case-control studies   1. >3 case-control studies |
|  |  |
| Limited evidence | Generally consistent findings in:   1. Single cohort study 2. 1 or 2 case-control studies 3. >2 cross-sectional studies or case series |
| Conflicting evidence | Inconsistent findings in <75% of the studies |
| No evidence | No studies could be found |
