# Supplementary material for: Inflammatory mediators in intra-abdominal sepsis or injury – a scoping review
Source: Crit Care. 2015 Oct 27;19:373. doi: 10.1186/s13054-015-1093-4 (PMC4623902; doi:10.1186/s13054-015-1093-4)
Supplement: Additional file 3: Table S3. — Clinical biomarkers. (DOCX 76 kb) [file 13054_2015_1093_MOESM3_ESM.docx]

**Table S3**. Summary of clinical studies of mediators as biomarkers in diagnosing complication or predicting outcomes of intra-abdominal sepsis/injury

| Study | Year | Design-  Sepsis/injury | No. of  patient | Mediator | Blood or P. fluid | Cutoff | Sen.  (%) | Spe.  (%) | Kinetics | Outcomes and interpretation |
| --- | --- | --- | --- | --- | --- | --- | --- | --- | --- | --- |
| Schentag et al.[21]  Duswald et al.[22]  Deby-Dupont et al.[23]  Mustard et al.[24]  Ingram et al.[25]  Dueholm et al.[26]  Damas et al.[27]  Hamilton et al.[28]  Ohzato et al.[29]  Fugger et al.[30]  Cabie et al.[31]  Gurleyik et al.[32]  Holzheimer et al.[33]  Kaufmann et al.[34]  Riche et al.[35]  Tang et al.[36]  Hammond et al.[37]  Frohlich et al.[38]  Berger et al.[39]  Gilliland et al.[40]  Groeneveld et al.[41]  Donati et al.[42]  Kato et al.[43]  Simovic et al.[44]  Holmberg et al.[45]  Pape et al.[46]  Erkasap et al.[47]  Holzheimer et al.[48]  Reith et al.[49]  Riche et al.[50]  Scheingraber et al.[51]  Bolke et al.[52]  Buttenschoen et al.[53]  Wiik et al.[54]  Gurleyik et al.[55]  Herwig et al.[56]  Lindberg et al.[57]  Ikuta et al.[58]  Yang et al.[59]  Latifi et al.[60]  Jansson et al.[61]  Chachkhiani et al.[62]  Karamarkovic et al.[63]  Mokart et al.[64]  Yamamoto et al.[65]  Sarbinowski et al.[66]  Kermarrec et al.[67]  Birchley et al.[68]  Haecker et al.[69]  Sack et al.[70]  Kowal-Vern et al.[71]  Dimopoulou et al.[72]  Novotny et al.[73]  Svoboda et al.[74]  Ilias et al.[75]  Cohen et al.[3]  Determann et al.[76]  Novotny et al.[77]  Schroeder et al.[78]  Buttenschoen et al.[79]  Hofer et al.[80]  Bingold et al.[81]  Brenner et al.[82]  Hranjec et al.[83]  Manganelli et al.[84]  Albayrak et al.[85]  Kumar et al.[86]  Ravishankaran et al.[87]  Tschaikowsky et al.[88]  Yamamoto et al.[89]  Nguyen-Vermillion et al.[90]  Steppan et al.[91]  Almeida et al.[92]  Azevedo et al.[93]  Benkoe et al.[94]  Bezmarevic  et al.[95]  Celik et al.[96]  Kimura et al.[97]  Witczak et al.[98]  Wu et al.[99]  Barbic et al.[100]  Jung B et al.[101]  Riché et al.[102]  Scepanovic et al.[103]  Stamataki et al.[104]  Yamanouchi et al.[105]  Zhu et al.[106] | 1984  1985  1987  1987  1988  1989  1992  1992  1992  1993  1993  1995  1995  1996  1996  1996  1996  1997  1997  1997  1997  1998  1998  1999  1999  1999  2000  2000  2000  2000  2001  2001  2001  2001  2002  2002  2002  2003  2003  2004  2004  2005  2005  2005  2005  2005  2005  2006  2006  2006  2006  2007  2007  2007  2007  2009  2009  2009  2009  2009  2009  2010  2010  2010  2010  2011  2011  2011  2011  2011  2011  2011  2012  2012  2012  2012  2012  2012  2012  2012  2013  2013  2013  2013  2013  2013  2013 | Cohort – Abdom. sepsis  Cohort – Abdom. sepsis  case control  - Abdom. sepsis or injury  cohort –  Abdom. sepsis  Cohort –  Abdom. infection  Case series -Abdom. infection  Case series  -Abdom. sepsis  Case series – Abdom. infection  Cohort –  Surgical injury  Cohort –  Abdom. sepsis  Case control  -Surgical injury, ischemia  Cohort –  Abdom. infection  Case series – Abdom. sepsis  Case control – Abdom. infection  Cohort –  Abdom. sepsis  Case control – Abdom. sepsis  Case control – Abdom. sepsis  Case series – Abdom. sepsis  Case series – Abdom. surgical injury  Case series  - surgical injury  Case series  - IR injury  Case series  -Surgical injury  Case series  - Surgical injury  Cohort –  - Abdom. Sepsis  Case control  - IR injury  Cohort –  Abdom. Injury  Cohort –  Abdom. Infection  Cohort –  Abdom. Sepsis  Case control  - abdom. Sepsis  Cohort –  Abdom. Sepsis  Case series – Abdom. Sepsis, TAC  Cohort –  Surgical injury  Case control  - surgical injury  RCT - G-CSF/ surgical injury  Case control  - abdom. Infection  Cohort –  Abdom. Sepsis  Case series – surgical injury  Case series – abdom sepsis  Cohort –  Abdom. Sepsis  Cohort –  Abdom. Sepsis  Case series – Abdom. surgical injury  Case control – Abdom. surgical injury vs. sepsis  Case control – Abdom. Sepsis vs. hernia repair  Cohort –  Abdom. Sepsis  Case control – Abdom. Sepsis (IBD)  Cohort –  Surgical injury  Case control – Abdom. Sepsis  Cohort –  Appendicitis  Case series– perforated appendicitis  Cohort –  appendicitis  Case series-  ACS  Cohort –  Surgical injury or infection  Cohort –  Abdom. sepsis  RCT – abdom. Sepsis  Case series – abdom. surgical injury  Cohort – Traumatic injury  Cohort –  Abdom. Sepsis  Cohort –  Abdom. Sepsis  RCT –  Abdom. Sepsis  Case series – surgical injury  Case control – Abdom. Sepsis  Case control – Abdom. Sepsis  Case control – Abdom. Sepsis  Cohort –  Injury or infection  Case series – Surgical injury  Case control – Abdom. Infection  Case control – Abdom. infection  Case control – Abdom. Sepsis  Case series – Abdom. sepsis  Cohort –  Surgical injury vs. infection  Case series – surgical injury  Case control – sepsis vs. surgical injury  Cohort –  anastomotic leakage  Case series – Sepsis  Cohort –  Abdom. Sepsis  Cohort –  Pancreatitis  Case control – surgical injury vs. sepsis  Case series – Abdom. Sepsis  Cohort –  Abdom. Sepsis  Case control – Acute appendicitis  Case series – surgical injury  Cohort –  Abdom. Sepsis  Case series – Abdom. Sepsis  Cohort –  Abdom. Infection  Case control –traumatic injury  Case control – sepsis vs. injury  Cohort –  surgical Ileus | 97  41  84 (+76 healthy control)  108  41  59  40  18  71  19  14 AAA + 7 internal carotid control  108  17  25 (+ 18 healthy control)  59  15 (+ 10 control)  11 (+ 9 control)  21  52  20  19  12  10  32  23 (+ 20 healthy control)  64  55  128  246 (+ 66 control)  52  19  52  20 (+ 10 healthy control)  30 (15 for one arm)  77 (10 healthy control)  24  47  28  46  19 infants  19  20 (+ 12 abdom. sepsis)  44 (+ 15 hernia repair)  50  50 (+ 50 surgical control)  50  29 (+ 13 healthy control)  80  13 children  211 children  29 burn patients  40  42  38 in PCT arm (34 control)  36  168  70  104  14 in PCT arm (13 routine)  20  101 (+ 28 surgical control)  16 (+ 16 surgical control)  87 (+ 28 surgical control)  1655  47  60 (+ 20 healthy control)  100 (+ 30 surgical control)  99 (+ 99 healthy control)  64  100  55 infants  132 (+ 18 healthy control)  173  28  40 infants  52  43 infants (+ 40 septic infants)  24  36  150 (50 healthy control)  41  101  66  156  64 (23 trauma pats)  66 (+ 6 healthy control)  100 | C-reactive protein (CRP)  Neutrophil elastase (E-a1-PI), CRP  TXB2,  6-keto-PGF1a  CRP  CRP, leukocytes (WBC)  CRP, WBC, neutrophil  APACHE II score, IL-1β, TNF, IL-6, CRP  IL-6, TNF-α, endotoxin (ET), T-cell function  IL-6, CRP  TNF-a, IL-6, APACH II, lactate  LPS, IL-6, TNF-a  CRP  ET, Elastase, neopterin, TNF-α, IL-6, IL-1  cICAM-1, CRP  TNF-α, IL-1, IL-6  TNF-a, IL-6, APACHE II  IL-1,IL-6, IL-8, TNF-α, ET  TNF-a, IL-8, E-a1-PI  IL-6, CRP, ET  TNF-α, IL-6, IL-10, IL-1ra  Neutrophil, C3a, TNF-a, IL-6, IL-8  IL-6, IL-8  IL-6, IL-8, IL-10  IL-6, IL-8, IL-10  IL-6, IL-10, MCP-1, sIL-2r, Fibrinogen  IL-6, ET, IgG and IgM antibodies to LPS  CRP, IL-6, WBC  IL-6, IL-8, APACHE II, MODS, MOF score  Procalcitonin(PCT), APACHE II, TNF-a, IL-6, neopterin, CRP  TNF-α, IL-1, IL-6  TNF-a, IL-6, IL-8, IL-10, CRP  ET, TxB2, LTC4, 6-keto-PGF1a, IL-6, CRP  ET, IL-6, CRP, α1-antitrypsin, α2-macroglobu.  IL-1, TNF-α, IL-6, IL-8, TGF-β, IL-10  IL-6  TNF-α, IL-1β, IL-6  PCT, CRP  IL-18, IL-10, IFN-γ, APACHE II  CRP  IL-6  CRP, TNF-α, IL-6, IL-10  IL-1β, IL-6, IL-8, TNFα, Leptin, IL-1ra, sIL-2R  Protein C, C5a, APACHE II  IL-6, PCT, CRP  IL-1β, IL-6, TNF-α  PCT, IL-6, C3a, CRP  TNF-α, TNF-α receptors, L-Selectin.  CRP, WBC, neutrophil  TNF-α, IL-6, IL-10,  CRP, IL-6, WBC, TNF-α, ET  IFN-g, TNF-α, IL-10, IL-6, IL-4, IL-2, AT  sTREM-1, TNF-a, IL-6, IL-8, IL-10  TNF-α, NO, cytochrome P450, ABT score  PCT, CRP, IL-6, TNF-α, AT-III, WBC  TNF-α, IL-6, IL-8, IL-10, T3  HMGB1, TNF-α, IL-6, vWF, PT, tPA, D-Dimers  sTREM-1  PCT  PCT, CRP  TNFα, IL-1β, IL-6, ET, CRP, α1-acid gp  CK-18, IL-6, sVCAM-1, sICAM-1, APACHE II, Lactate  IL-6, IL-10, IL-22  IL-6, sICAM-1, sVCAM-1, MIF, APACHE II  IL-1, -2, -4, -6, -8, -10, -12, TNF-α  HMGB1, IL-6  HMGB1  CRP  IL-6, CRP, lactate  PCT, IL-6, CRP  IL-1β, IL-6, TNF-a  CRP, IL-6, IL-1β, IL-8, IL-10, TNF-a  IL-6, ICAM-1, VCAM-1, Syndecan-1, Heparan  CRP  PCT  IL-8, CRP  CRP, PCT, IAP  IL-6, CRP  IL-15, creatinine, PaO2/FiO2  CRP  HMGB1  IL-6, PCT, CRP  PCT  IL-1, TNF-a, IL-6, IL-10, IFNγ  CRP, WBC  S100B, IL-6, lactate  mtDNA (mitochondrial DNA)  PCT, CRP, TNF-α | Blood  Blood  Blood  Blood  Blood  Blood  Blood  Blood  Blood  Blood  Blood  Blood  Blood and P. fluid  Blood  Blood  Blood  Blood  Blood, and P. fluid  Blood  Blood  Blood  Blood  Blood  Blood  Blood  Blood  Blood  Blood  Blood  Blood  Blood, P. fluid  Blood  Blood  Blood, P. fluid, wound fluid  Blood  P. fluid  Blood  P. fluid  Blood  Blood  Blood, P. fluid  Blood  Blood  Blood  Blood, P. fluid  Blood  Blood, P. fluid  Blood  Blood, P. fluid  Blood  Blood, P. fluid  Blood  Blood  Blood  Blood  Blood  Blood, P. fluid  Blood  Blood  Blood  Blood  Blood  Blood  Blood  Blood, monocyte  Blood  Blood  Blood  Blood  P. fluid  Blood  Blood  Blood  Blood  Blood  Blood  Blood  Blood  Blood  Blood  Blood  Blood  Blood, P. fluid  Blood  Blood  Blood  P. fluid | 11 mg/dl  86.5 ng/ml (E-α1-PI);  10 mg/dl (CRP)  140 pg/ml (TXB2); 120 pg/ml (6-keto-PGF1a)  (1) on POD 3, CRP> 80% on POD 2;  (2) after POD 4, CRP> 15 mg/L  10 mg/L (CRP)  6 mg/L (CRP)  −  −  −  −  −  10 mg/L  −  500 ng/ml (ICAM-1), 120 mg/L (CRP)  −  −  −  −  −  −  −  −  −  −  −  −  8 mg/L (CRP),  60 pg/ml (IL-6), 10x10^9^/L (WBC)  lL-6 (>1000 pg/ml), IL-8 (>70 pg/ml) APACHE II (>10)  −  −  −  −  −  −  100 pg/ml  −  −  −  28 mg/L  500 pg/ml  −  −  66% of normal activity  1.1 ng/ml (PCT), 310 pg/ml (IL-6), 93 mg/L (CRP)  −  −  −  −  IL- 6 <15 pg/ml, TNF-α<20 pg/ml  IL-6, 12.2 pg/ml, WBC 14.3x10^9^ CRP 11 mg/L  −  −  −  2 ng/ml (PCT)  −  −  160 pg/ml  1.03:1.0 (POD1:POD2)  1. ≤1 ng/ml  2. 25-35% of initial value  −  −  −  −  −  −  25 ng/ml  1) 12 mg/L  2) 24 mg/L  2760 pg/ml (IL-6), 205 mg/L (CRP), < 3.9mM (lactate)  POB<50% (PCT)  −  −  −  140 mg/L  −  449 pg/ml (IL-8)  120 mg/L (CRP),  0.25 ng/ml (PCT)  −  −  150 mg/L  −  100 pg/mL (IL-6), 95.4 mg/L (CRP)  0.5 ng/ml (or 20% of peak value)  −  135 mg/L (CRP)  2.1 µg/ml (S100B)  −  − | −  −  −  63%  −  100%  −  −  −  −  −  93%  −  75% (ICAM-1), 67% (CRP)  −  −  −  −  −  −  −  −  −  −  −  −  96% (CRP), 94% (WBC), 33% (IL-6)  84% (for mortality)  −  −  −  −  −  −  84%  −  −  −  100%  −  −  −  80%  90% (IL-6), 81% (PCT), 63% (CRP)  −  −  −  −  −  IL-6 75.8%, WBC 75.8%, CRP 69.7%  −  −  −  −  −  −  88%  95%  −  −  −  −  −  −  −  72%  1) 94%  2) 42%  87% (IL-6), 57% (CRP), 100% (lac)  97% (PCT)  −  −  −  78%  −  82.8% (IL-8)  75% (CRP),  86% (PCT)  −  −  96%  −  87.7% (IL-6), 87% (CRP)  48%  −  73%  86%  −  − | −  −  −  82%  −  32%  −  −  −  −  −  80%  −  85% (ICAM-1)  −  −  −  −  −  −  −  −  −  −  −  −  87% (CRP), 89% (WBC)83% (IL-6)  90% ( for mortality)  −  −  −  −  −  −  46%  −  −  −  54%  −  −  −  88%  58% (IL-6), 72% (PCT), 72% (CRP)  −  −  −  −  −  IL-6 100%, WBC 62.5%, CRP 100%  −  −  −  −  −  −  67%  63%  −  −  −  −  −  −  −  73%  1) 60%,  2) 100  81% (IL-6), 46% (CRP) 83% (lac)  −  −  −  −  86%  −  81.8% (IL-8)  86% (CRP),  63% (PCT)  −  −  79%  −  60.6% (IL-6), 67% (CRP)  94%  −  73%  99%  −  − | Yes  Yes  Yes  Yes  −  −  Yes  Yes  Yes  Yes  Yes  −  Yes  −  Yes  Yes  Yes  Yes  Yes  Yes  Yes  Yes  Yes  Yes  Yes  Yes  −  −  Yes  Yes  Yes  Yes  Yes  Yes  −  Yes  Yes  −  −  Yes  Yes  Yes  Yes  −  −  Yes  Yes  −  Yes  −  Yes  Yes  Yes  Yes  Yes  −  Yes  Yes  Yes  Yes  Yes  −  Yes  −  Yes  −  −  −  Yes  Yes  Yes  Yes  Yes  Yes  −  −  Yes  Yes  Yes  −  Yes  Yes  −  Yes  Yes  Yes  Yes | CRP levels peaked on POD3, fell below 10 mg/dl on POD5 in patients who had effective antibiotics therapy. Persistent CRP levels of > 10 mg/dl indicated abscess formation or continued infection.  The E-α1-PI levels of the patients without pre- or postoperative infection were in the normal range; rose to more than 10 times when septicemia was confirmed, returning to normal during recovery; but remained significantly elevated until death if not recovery.  In patients with ARDS, the median values of TXB2 and 6-keto-PGF1α were 575 pg/ml and 122 pg/ml, respectively. In patients without ARDS, those values were 140 pg/ml and 25 pg/ml, respectively, (p < 0.05). High TXB2 and 6-keto-PGF1α values were particularly related to sepsis in abdominal surgery patients (p < 0.05) and in multiple injured patients (p < 0.01).  The overall accuracy rate for detecting infection was 75%. A normal CRP response to surgery without a secondary rise might exclude a postoperative septic complication, as demonstrated by the negative predictive value of 78%. The positive predictive value of this test was found to be low (69%).  The mean CRP level on admission in patients with abdominal infection was 57.0± 10.8 mg/L, significantly different (p<0.05) from patients without infection (27.0± 13.7 mg/L), or those with ‘non-specific abdominal pain’. The values of WBC were not significantly different.  The most efficient test combination predicting a patient free from appendicitis was WBC count < 9 X 10^9^/L, neutrophils < 75%, and CRP < 6 mg/L on admission, demonstrating a predictive value of a negative result of 100% with a specificity of 32%.  The mortality rate increased significantly in the group of patients who presented with IL-6 serum level above 1000 pg/ml. Thus, IL-6 was suggested to be a good marker of severity during abdominal bacterial infection. TNFα level was above100 pg/ml during septic shock. There was a direct correlation between IL-6 and CRP serum levels.  IL-6 was elevated due to the surgical trauma associated with the re-operations. An anergic immune status with depressed T-lymphocyte function in patients with poor prognosis. Attempts to neutralize mediators might have further adverse effects on the immune system in the late stages of intraabdominal infections.  IL-6 levels reached a maximum at least 12 to 24 hours earlier than the CRP level resulted from surgical trauma. The postoperative CRP correlated with IL-6 in patients with normal liver function. IL-6 was correlated with the length of the operation and blood loss, but CRP showed no significant correlation with these factors.  Perioperative concentrations of both TNFα and IL-6 were significantly higher in the 4 patients with septic shock than sepsis or non-septic patients. The preoperative APACHE II score correlated with the increased TNFα concentration (r = 0.5, p < 0.001), and plasma lactate concentration with that of IL-6 (r = 0.7, p = 0.003).  LPS and TNFα were not detected in control patients. TNFα levels were higher in portal than in systemic blood in patients with AAA plus aortic clamping after reperfusion. Levels of IL-6 were similar in the two groups, with a peak on the day following surgery. Aortic clamping and reperfusion led to similar levels of portal and systemic LPS.  In 90 patients with acute appendicitis, serum CRP concentrations were elevated in 87 patients and normal in 3 patients. Of 18 patients with a normal appendix, serum CRP  concentrations were normal in 16 patients and slightly elevated (8.4 and 12.6 mg/L) in 2 patients. The accuracy of pre-operation CRP to predict acute appendicitis was 91%.  Plasma levels of endotoxin, elastase, neopterin, TNFα, and IL-6 were higher in the nonsurvivors than in the survivors. Peritoneal levels of endotoxin, TNFα, elastase, and IL-6 were 7-fold, 19-fold, 239-fold, and 993-fold higher, respectively, in the peritoneal exudate than in the plasma. The systemic and local inflammatory response persisted during relaparotomy in the 6 nonsurvivors.  Patients with necrotizing pancreatitis had plasma cICAM-1 concentrations of 729 ± 106 ng/ml, significantly different from patients with edematous disease (367 ± 48) and controls. Plasma cICAM-1 levels were not significantly different between controls and patients with mild pancreatitis. Plasma ICAM-1 could serve as a marker of a severe clinical course of pancreatitis. C-reactive protein was not sensitive to predict severity of acute pancreatitis.  No difference was observed between survivors and nonsurvivors for serum IL-1 and IL-6 levels. Mean serum TNFα level tended to be higher in survivors than in nonsurvivors (565 +/-1325 versus 94 +/- 69 pg/ml) on post sepsis day 1.  In septic patients, the mean APACHE II score increased from 20.3 to 24.5 (p <0.05) 24 hrs after surgery. The post-surgery APACHE II score correlated significantly with intraoperative peak TNF-alpha (r2 = 0.32, p < 0.05) and peak IL-6 (r2 = 0.38, p < 0.01) concentrations. Only IL-6 concentration increased postoperatively, which was related to poor outcome.  IL-6, TNF-α, and IL-8 shown higher values in the sepsis group than the control group pre-operatively. IL-6 increased after sepsis, it further increased after surgery injury.  The average pre-operative levels of TNFα, IL-8, and E-α1-PI were 100 times more in peritoneal exudate than in plasma. Surgical procedures reduced the intra-abdominal concentrations of cytokines. There were no significant differences in the intra-abdominal or plasma levels of cytokines, or PMN-E-α1-PI between survivors and non-survivors.  IL-6 levels began to increase at the end of abdominal surgery with a maximum before wound closure (299 and 511pg/mL, respectively), IL-6 declined on post-operation day 1, and back to baseline on post-operation day 3 in the case of no complications. CRP values rise on post-operation day 1, and the highest levels were recorded on day 2 after major surgery.  All cytokine levels were very low or under detectable at baseline or during hysterectomy. TNF-α levels did not increase post-operation. Other cytokines increased 4 h post-operation then declined quickly. IL-6 level remained high for 24 h post-operatively.  Aortic artery surgery and ischemia and reperfusion (I/R) activates complement, releases cytokines (except for TNF-α), and induces neutrophil recruitment and degranulation.  The highest mean levels of IL-6 were observed 2 h after the skin incision and at the end of abdominal surgery. IL-6 levels remained above normal until 48 h after surgery. Huge range of IL-6 (123 to 5000 pg/ml) among patients was observed. IL-8 values were normal all time.  Levels of the three cytokines increased after the start of abdominal surgery, peaking at the end of operation (145.1 pg/ml for IL-6, 46.0 pg/ml for IL-8, and 15.2 pg/ml for IL-10). IL-10 remained elevated for one day and IL-6 remained high for 2 days after surgery.  Patients with severe pancreatitis had significantly elevated plasma concentrations of IL-10 and IL-6, but not IL-8, compared with patients with mild pancreatitis. IL-10 plasma concentration was significantly elevated on day 5 in patients with severe pancreatitis who died compared with survivors (p < 0.027).  During aortic clamping, IL-6, IL-10 and MCP-1 increased significantly (p<0.001, p<0.01 and p<0.05 respectively) while sIL-2R and fibrinogen decreased significantly (p<0.001 for both). After aortic declamping, IL-6, IL-10 and MCP-1 had further significant increases compared with levels during aortic clamping while sIL-2R and fibrinogen had a further decrease.  In the nonsurvivors, IL-6 levels were significantly elevated compared with surviving patients early after trauma, then decreased on days 7, 9, 11, 15, 17, and 19. In the survivors, IL-6 levels were significantly elevated compared with normal controls on days 5 and 7, and the IgG and IgM antibody levels against LPS increased during the second week. No antibody increase was seen in the nonsurvivors.  There were significantly higher levels of WBC counts and CRP levels in patients with acute, gangrenous and perforated appendix. The test combination of WBC or CRP gave positive results with 100% sensitivity. IL-6 concentration was significantly higher only in patients with perforated appendicitis. As a result, measurement of the CRP levels and WBC has an additional diagnostic value on the diagnosis of the acute appendicitis.  APACHE II (>10), lL-6 (>1000 pg/ml), IL-8 (>70 pg/ml), were significantly different between survivors and non-survivors and patients with/without severe complications by univariate analysis. By multivariate analysis only MOF, MODS score, IL-6, comorbidity predicted complications with a sensitivity of 82 % and a specificity of 87%; only APACHE II score, IL-8 predicted death (sensitivity 84 %; specificity 90 %).  The 59 nonsurvivors had initial mean PCT value of 4.2 ng/ml, raised to 13.8 ng/ml on day 1 to day 4 and 13.2 ng/ml at the time of death. The 187 survivors had initial mean PCT of 2.1 ng/ml, raised to 4.9 ng/ml on day 1 to day 4, and then a reduction to 0.4 ng/ml. PCT showed significant differences between survivors and patients who died (p < 0.05 on days 1 and 4 and at the endpoint); CRP did not. TNFα and IL-6 concentrations declined if no infection.  The median cytokine serum concentrations on day 1 of septic shock were: TNF-α, 90 pg/mL (6-4663 pg/mL); IL-6, 5000 pg/mL (44-5100 pg/mL). TNF-α and IL-6 levels decreased significantly between the first and third days. IL-6 serum levels tended to remain higher in the nonsurvivors group (p =0.057).  Serum IL-6 values increased from 2,135 pg/mL to 3,724 pg/mL after open abdominal lavage, and returned to 2,342 pg/mL 24 hours postoperatively. Peritoneal IL-6 levels were 100- to 1,000-fold higher, TNF-α and IL-8 levels were about 10- to 300-fold higher than serum values. IL-10 concentrations showed no changes in the course of abdominal lavage. There were no differences in cytokine concentrations between survivors and nonsurvivors.  Plasma mediators of the arachidonic acid cascade (LTC4 , PGF1α,and TxB2) were only elevated during and following abdominal surgery. IL-6 plasma levels peaked before wound closure. CRP levels peaked on post-operation day 2. Plasma levels of endotoxin significantly correlate with the severity of the surgical Intervention.  Endotoxemia peaked 1 hour after surgery at 0.16 EU/mL, and remained raised 24 hours later and decreased after 48 hours. Two hours after starting the operation, IL-6 concentration was significantly increased (470 pg/mL) and remained on this level for 24 hours. CRP level peaked 48 hours after the operation (210 mg/L). α1-Antitrypsin and α2-macroglobulin decreased during operation and increased thereafter.  Peritoneal cytokines levels showed 10 – 1000 times higher than blood levels. Perioperative filgrastim (r-metHu-G-CSF) had no effect on the cytokine levels in the first 24 h post-operation.  High false negative and positive rates of the serum IL-6 test for diagnosing appendicitis. Serum IL-6 results are not useful for preventing negative laparotomies.  Peritoneal IL-6 levels raised on POD1 (162,500 pg/ml) in patients with anastomotic leakages and increased until day 9 (144,500 pg/ml). IL-6 levels in patients without complications were significantly lower on POD1 (27,940 pg/ml) and kept declining.  PCT declines from POD1 and reaches half its initial values on POD2, whereas CRP increases in the first 48 h and reaches half its maximum value on POD5 in 47 patients with a normal postoperative course after major abdominal surgery.  Higher APACHE II scores (14.1 versus 7.8, P= 0.0073), higher incidence of SIRS (88% versus 35%, P = 0.0329), and peritoneal IL-18 concentrations distinguished patients who developed organ failure from those who did not. No significant differences in the peritoneal IL-10 or IFN-gamma concentrations between these patients.  The diagnostic sensitivity for C-reactive protein test was 100%.The specificity was 54.2%. The overall accuracy was low (76%) to predict abdominal infection on admission.  Preoperatively there was a wide range from 48 to 132546 pg/ml. Persistent IL-6 levels greater than 500 pg/ml may be useful in identifying pediatric patients with prolonged hospitalization and greater mortality.  Significantly higher peritoneal TNF-α, IL-6, and IL-10 values were recorded compared with systemic values, except for CRP. IL-6 values peaked between 3 and 9 hours after surgery and then declined.  Serum values of IL-6, TNF-α, and IL-8 peaked 24 h to 36 h after colorectal surgery (1125, 205, and 520 pg/ml, respectively), then declined during the next 48 to 72 h. These peak values were significantly lower than the values in the septic group (3258, 415, and 916 pg/ml, respectively). No differences were recorded between septic and no-septic groups in terms of IL-1β, Leptin, IL-1ra, and sIL-2R.  Preoperative protein C level was significantly lower in patients developed septic shock with lethal outcome, than in those survived (p = 0.0001). Protein C was of excellent predictive value and achieved a sensitivity of 80% and a specificity of 87.5% in discriminating survivors from non-survivors within the first 48 hours of the study (AUC-0.917; p < 0.001).  Sixteen patients developed septic complications during the first five postoperative days (group 1) and 34 patients did not (group 2). On day 1, PCT and IL-6 levels were significantly higher in group 1 (P = 0.003 and 0.006, respectively) but CRP levels were similar. PCT and IL-6 appear to be early markers of subsequent postoperative sepsis in patients undergoing major surgery. IL-6 yielded higher discriminative value (AUC of 0.82) than PCT (AUC=0.75).  The peritoneal IL-1β, IL-6, and TNF-α levels were higher than the plasma levels, and significantly higher in IBD patients with sepsis than in colorectal patients and acute appendicitis group.  During the early postoperative period after uncomplicated major abdominal surgery, SIRS was reflected in an increase in plasma PCT and C3a concentrations only. All mediator values were very low (CRP peak 51.5 mg/L; IL-6, 35.5 pg/ml; PCT, 0.53 ng/ml). No significant difference between SIRS and non-SIRS groups.  Plasma levels of TNF-α, TNFRI, and TNFRII were significantly higher in patients with diffuse peritonitis than in healthy controls. Peritoneal levels of the 3 mediators were 4 times higher than the plasma levels in patients.  In patients required laparotomy for suspected acute appendicitis, white cell count and neutrophil count distinguished acute appendicitis from normal, whereas CRP did not.  The peritoneal cytokine levels were more than 100 to 1000 times greater than plasma levels. High TNF-α levels in the peritoneal fluid were associated with clinical improvement. Compartmentalization of infection by the peritoneum prevents further systemic reactions.  Distinguishing early appendicitis from phlegmonous or perforative appendicitis by pre-operation CRP or IL-6 levels. WBC count correlated significantly to the severity of appendiceal inflammation. Serum endotoxin level was elevated in cases with perforation. ESR and TNF-α showed a poor correlation to appendiceal inflammation.  Plasma levels of anti-thrombin were lower and peritoneal levels of IL-10, IL-6, IL-4 were higher in nonsurvivors, compared to survivors. Other cytokines were not different between the two compartments.  Compared to baseline levels, TNF-a, IL-8, and IL-10 elevated on day 1; IL-6 elevated on days 1 and 2; post-operatively. Soluble triggering receptor expressed on myeloid cells-1 (sTREM-1) did not raised. There were no differences in cytokine concentrations between patients who exhibited post-operative infection complications and those who did not.  TNF-α serum levels differed significantly between survivors and nonsurvivors preoperatively (P < 0.045) and at late stages of the septic course (P <0.001). ABT scores and Cytochrome P450 activity differed significantly between survivors and nonsurvivors. Septic liver failure is linked to the induction of NO synthesis in systemic inflammation.  The hospital mortality was 26% in PCT monitoring group versus 38% in control group (p=0.28); the ICU days were 16.1 versus 19.4 in control (p=0.09) and ventilated days were 10.3 versus 13.9 in control (p=0.08). No significant difference for the outcomes between the two groups was reached by using the PCT value as a guide for imaging and interventions.  IL-6 and IL-8 peaked on post-operation day 1 whereas IL-10 peaked immediately postoperatively (IL-6, 379; IL-8, 20.3; IL-10, 36.3 pg/ml). The increase of TNF-α was not significant. Triiodothyronine (T3) was the highest before surgery.  Plasma levels of HMGB1 correlate with injury severity and shock; and correlate with early post-traumatic coagulopathy and other markers of systemic inflammation. Early HMGB1 elevation is associated with increased morbidity and mortality in trauma patients.  PF sTREM-1 levels were higher than systemic levels (P < 0.001), but no significant differences between survivors and non-survivors. On POD2 and 3, patients with ongoing infection had significantly higher PF sTREM-1 levels compared to patients without infection.  PCT ratio between POD1 and 2 greater than 1.03 suggested successful eradication of the septic focus with no further invasive measures necessary. Ratio less than 1.03 should critically reevaluate patients for ongoing infection.  The length of antibiotic treatment was 6.6±1.1 days in the PCT-guided group versus 8.3±0.7 days in the control group. No negative effects on outcome were observed.  The plasma levels of endotoxin, TNFα and IL-6 peaked 2 h postoperatively. Plasma CRP and α1-acid glycoprotein peaked 48 h postoperatively. Plasma IL-1 was not altered after surgery.  The APACHE II score, lactate, levels of total CK-18 and CK-18 fragments were significantly higher at the time of diagnosis of sepsis in nonsurvivors. Levels of sICAM-1, sVCAM-1, and IL-6 were comparable between nonsurvivors and survivors.  IL-6 and IL-10 in sepsis patients were higher than in non-sepsis surgical controls. No difference in systemic IL-22 was found between sepsis patients and healthy control.  In the nonsurviving subgroup, higher IL-6 levels (P = 0.083 vs. surviving subgroup), APACHE II-score (30.5 vs. 26 survivors; P = 0.003), and MIF at the onset of sepsis were recorded; sICAM-1), sVCAM-1, and CRP were not significantly different between the two subgroups.  Nonsurviving trauma patients had higher IL-4, -6, -8 and TNF-α, while IL-4 was the most significant predictor of death. In infected patients, nonsurvivors had higher admission levels of IL-2, -8, -10, no cytokine independently predicted death.  HMGB1 in monocytes and patients’ blood was significantly higher post-surgery. HMGB1 induced monocytes to release IL-6 *in vitro*.  The average HMGB-1 level of the patients with acute appendicitis was 36.92 ng/ml while the value of the healthy group was 21.71 ng/ml, p = 0.001. HMGB-1 might be useful in the diagnosis of acute appendicitis.  The accuracy of using CRP to diagnose acute appendicitis was low.  The AUC for serum lactate (0.922), IL-6 (0.912), and CRP (0.719) in differentiating between patients with severe sepsis and those with sepsis were reported. Combined use of serum lactate and IL-6 aided in establishing both the severity of sepsis and the prognosis of acute abdomen.  No significant difference in baseline APACHE II scores or plasma concentrations of PCT, IL-6, and CRP was found between survivors and nonsurvivors. PCT and IL-6 decreased from day 1 to 14 in survivors, but increased in non-survivors. At day 7, PCT-POB less than 50% was an independent determinant for survival.  On POD1 & 2, the IL-1β, IL-6 and TNF-α levels were not significantly different between patients who developed or did not peritonitis. On POD3, these cytokines were significantly higher in patients who developed peritonitis.  In the absence of infection, CRP levels peaked on POD2, declining by POD3. IL-6 levels peaked on POD1, correlated with CRP peak (r = 0.398, P =0.004).  Syndecan-1, heparan sulfate, and IL-6 were markedly higher in the sepsis group and the surgery group. VCAM-1 and ICAM-1 were significantly higher in the sepsis group.  On POD2, serum CRP was 187 mg/L in leakage group and 132 mg/L in non-leakage group (p=0.001). On POD 3, those values were 201 mg/L and 105 mg/L (p < 0.001). Daily CRP measurements seemed useful to predict anastomotic leakage after colorectal surgery.  The 24-h procalcitonin clearance (PCT-c) was significantly higher in the group of survivors (p = 0.028). Persistently high PCT in plasma was associated with a significant increase in mortality in patients with severe sepsis.  Preoperative serum levels of CRP failed to predict intestinal involvement in infants with necrotic enterocolitis (NEC). Serum levels of IL-8 were able to predict intestinal involvement (OR, 1.74; P <0 .001, AUC = 0.81).  There was significant correlation between PCT and intra-abdominal pressure (IAP) values measured at 24 h of admission of acute pancreatitis, and between maximal PCT and IAP values. CRP, PCT and IAP may be used as markers to predict acute pancreatitis progression.  In the surgery group, IL-6 peaked on POD2 and CRP increased on POD4, then decreased to preoperative levels. In septic neonates, IL-6 increased on the first day of sepsis, CRP levels were low.  A positive correlation on POD1 between IL-15 serum levels and the duration of SIRS (R = 0.50, P<0.05), the creatinine levels (R=0.48, P<0.05); and a negative correlation on POD3 between IL-15 levels and the PaO2/FiO2 (R= -0.69, P<0.01).  Postoperative septic complications can be anticipated if CRP on POD5 is higher than 1/2 of the maximum CRP concentration on POD2 or POD3, or CRP > 150 mg/L on POD3. But the severity of the complication cannot be projected based on CRP level.  HMGB1 concentrations were10.97 ng/ml in acute simple appendicitis group, 5.47 ng/ml in healthy controls (P <0.01), and 18.08 ng/ml in gangrenous appendicitis.  IL-6 > 100.4 pg/mL at POD1 could predict SIRS with a sensitivity of 87.75% and specificity of 60.61%. CRP value of 95.4 mg/L at POD3 yielded sensitivity of 87.5% and specificity of 66.67% in detecting SIRS. PCT was not accurate in diagnosis of SIRS after abdominal surgery.  PCT levels upon ICU admission predicted subsequent failure of index laparotomy (P = 0.04). But a PCT threshold of 0.5 ng/ml or 80% reduction from its peak was not accurate to predict treatment response.  The peritoneal fluid cytokines’ levels were from 5 (2 to 21) (IFNγ) to 1310 (145 to 3888) (IL-1) folds higher than the plasma levels. Plasma TNFα, IL-6, IFN and IL-10 were higher in patients with shock versus no shock and in nonsurvivors versus survivors (P ≤0.03). Peritoneal IL-10 was higher in patients who survived (1505 (450 to 3130) versus 102 (9 to 710) pg/ml; P = 0.04).  CRP was significantly higher every day during the first 7 post-abdominal surgery days in patients who developed anastomotic leakage compared with those who did not. Patients with CRP values higher than 135 mg/L on POD 3 require an intensive search for infective complications, particularly anastomotic leakage.  In predicting death, preoperative S100B yielded ROC curve area of 0.86 for trauma patients. Severe hemorrhage without brain injury is associated with increased serum levels of S100B, which correlates with IL-6.  Concentrations of mtDNA peaked on day 1 in patients with trauma, whereas they increased on day 1 and remained constant until day 5 in patients with sepsis. The mtDNA levels on day 1 were significantly higher in the two nonsurvivors compared with survivors of trauma (P < 0.05) but not with the severity of injury.  PCT peaked on POD1 (0.2 ng/ml) in non-ileus patients, declined on POD3; CRP level peaked on POD3 (28 mg/L), declined on POD5. |

Sen. = sensitivity. Spe. = specificity. P. fluid = peritoneal fluid.

**Abbreviations:**  AAA, abdominal aortic aneurysm; ABT, aminopyrine breath test; APACHE II, acute physiology and chronic health evaluation II; CK-18, cytokeratin-18; CRP, C-reactive protein; E-a1-PI, neutrophil elastase-α1-proteinase inhibitor complex; ET, endotoxin; G-CSF, granulocyte colony-stimulating factor; HMGB1, high mobility group box nuclear protein 1; IFN-γ, interferon-γ; ICAM-1, intercellular adhesion molecule 1; IL, interleukin; IL-1ra, IL-1 receptor antagonist; IR, ischemia and reperfusion; 6-keto-PGF1a, 6-keto-prostaglandin F1a; LPS, lipopolysaccharide; LTC4, leucotriene-C4; MCP-1, monocyte chemoattractant protein-1; MIF, macrophage migration inhibitory factor; MODS, multi-organ dysfunction syndrome; MOF, multi-organ failure; mtDNA, mitochondrial DNA; NO, nitric oxide; PCT, procalcitonin; SICU, surgical intensive care unit; SOFA, sequential organ failure assessment; sTREM-1, soluble triggering receptor expressed on myeloid cells-1; sVCAM-1, soluble vascular cell adhesion molecule 1; TGF-β, transforming growth factor β; TNF, tumor necrosis factor; tPA, tissue plasminogen activator; TXB2, thromboxane B2; vWF, von Willebrand factor.
