# Supplementary material for: Inflammatory mediators in intra-abdominal sepsis or injury – a scoping review
Source: Crit Care. 2015 Oct 27;19:373. doi: 10.1186/s13054-015-1093-4 (PMC4623902; doi:10.1186/s13054-015-1093-4)
Supplement: Additional file 4: Table S4. — Clinical mechanism. (DOCX 19 kb) [file 13054_2015_1093_MOESM4_ESM.docx]

**Table S4**. Summary of clinical mechanistic studies of mediators in intra-abdominal sepsis/injury

| Study | Year | Design-  Sepsis/injury | No. of  patient | Mediators | Blood or P. fluid | Type of  mechanism | Outcomes and interpretation |
| --- | --- | --- | --- | --- | --- | --- | --- |
| Moore et al [107]  Holzer et al [108]  Poeze et al [109]  Riese et al [110]  Adembri et al [111]  Boldt et al [112]  Sperry et al [113]  van Till et al [114] | 1991  2002  2002  2002  2004  2004  2008  2010 | Case series –  Traumatic injury  Case control – Abdom. Sepsis  Cohort –  Surgical injury  Case control – Abdom. Sepsis  Case control – surgical injury  RCT – NS:RL:HES  Cohort –  Traumatic injury  Case control – Abdom. sepsis | 20  17 (+ 7 control)  26  22 (+ 66 controls)  12 AAA (+ 12 controls)  66 (22 in each arm)  80  39 (+ 8 controls) | IL-6, TNF-α, C3a, ET, bacteremia  Neutrophils (phagocytosis)  IL-6, IL-8, sICAM-1, STNF-R55, sTNF-R75  IL-6, MCP-1  IL-6  CRP, IL-6, IL-8, WBC, sICAM-1  IL-1β, IL-6, TNF-α, IL-8, IL-10  IL-8, MCP-1, nucleosome, E-α1-PI, WBC | Blood (portal, systemic)  Blood, P. fluid  Blood (hepatic, systemic)  Blood  Blood  Blood  Blood  Blood, P. fluid, BAFL | Gut bacterial translocation  Phagocytosis of E Coli  Cytokines in hepatic dysfunction  Cytokine response  IL-6 in pulmonary dysfunction  inflammation and endothelial activation  Gender response to injury  Mediators in 3 compartments | Endotoxin could not be detected in portal or systemic blood at 48 h. No difference in portal and systemic blood levels of C3a, TNFα, and IL-6 in those patients who developed MOF or not. Portal or systemic bacteremia was not confirmed within POD 5.  The percentage of peritoneal neutrophils which engulfed E. coli bacteria was depressed in patients with peritonitis when compared to that of elective surgical patients, or compared to circulating PMNs (p < 0.05). The depression of phagocytosis correlates well with a decrease in CD16 receptors on neutrophils.  Mean arterial levels of IL-8, s-ICAM-1, s-TNF-R75, and s-TNF-R55 were significantly higher in patients with hepatic dysfunction.  The mean serum MCP-1 was 216 pg/mL in uninfected patients, 551 pg/mL in patients with peritonitis who survived, and 752 pg/mL in those who died (P < 0.05). The serum IL-6 levels were significantly higher in patients with peritonitis who survived (1203 pg/mL) and who died (752 pg/mL) compared with uninfected patients (8 pg/mL; P < 0.001).  Plasma IL-6 peaked at 6 and 12 h after reperfusion in aortic abdominal aneurysm surgery, which was followed (12 and 24 h after surgery) by a significant reduction of PaO2/FIO2 index and PaO2/PAO2 ratios.  In elderly patients after major abdominal surgery, markers of inflammation and endothelial injury/ activation were significantly higher after crystalloid (normal saline) than after HES 130/0.4-based volume replacement regimens.  IL-6 serum levels were statistically higher in males than females after injury (p = 0.008). No significant difference in IL-1β, TNF-α, IL-8, and IL-10 serum levels was found when compared across gender over time. Males had a significantly higher rate of MOF.  Levels of IL-8, MCP-1, MPO, and elastase were the highest in peritoneal fluid (PF > BALF > plasma). BALF MCP-1 and MPO levels in peritonitis patients were higher compared with surgical controls. Severe peritonitis produces an early pulmonary expression of chemoattractants to the lungs. |

**Abbreviations**: AAA, abdominal aortic aneurysm; BALF, bronchoalveolar lavage fluid; CRP, C-reactive protein; E-a1-PI, neutrophil elastase-α1-proteinase inhibitor complex; ET, endotoxin; HES, Hydroxyethyl starch; ICAM-1, intercellular adhesion molecule 1; IL, interleukin; ISS, injury severity score; MCP-1, monocyte chemoattractant protein-1; MPO, myeloperoxidase; P. fluid, peritoneal fluid; sTNF-R, soluble-TNF-receptors; TNF, tumor necrosis factor.
