# Supplementary material for: Inflammatory mediators in intra-abdominal sepsis or injury – a scoping review
Source: Crit Care. 2015 Oct 27;19:373. doi: 10.1186/s13054-015-1093-4 (PMC4623902; doi:10.1186/s13054-015-1093-4)
Supplement: Additional file 5: Table S5. — Clinical intervention. (DOCX 22 kb) [file 13054_2015_1093_MOESM5_ESM.docx]

**Table S5**. Summary of clinical intervention studies of mediators in intra-abdominal sepsis/injury

| Study | Year | Design-  Sepsis/injury | No. of  patient | Mediators | Blood or P. fluid | Type of  intervention | Outcomes and interpretation |
| --- | --- | --- | --- | --- | --- | --- | --- |
| Chen et al. [115]  Matsuno et al. [116]  Schmidt et al. [117]  Schwarz et al. [118]  Bakker et al. [119]  Schietroma et al. [120]  Schietroma et al. [121]  Sista et al. [122]  Kirkpatrick et al. [123] | 2001  2001  2007  2007  2012  2012  2013  2013  2015  (epub2014) | Case control – surgical injury  Case series –  Abdom. Sepsis  RCT –  Hepatic resection  Cohort –  Abdom. Sepsis  RCT –  Infected pancreatitis  RCT –  Perforated appendicitis  RCT –  Perforated peptic ulcer  RCT –  Cholecystitis with sepsis  RCT –  Abdom. Sepsis or injury | 35 in treated (47 in control)  25  10 in steroid arm (10 controls)  50  10 in surgical arm (10 in endoscopy)  74 open surgery (73 in laparoscopy)  58 open repair (57 laparoscopic repair)  22 open surgery (23 laparoscopy)  23 with AbThera™  (22 with Barker’s pack) | CRP, IL-6, TNF-α  IL-6, IL-1ra, ICAM-1, PAI-1  TNF-α, IL-6, IL-8, IL-10, CRP  ET, ENC, IL-6, TNF-a, CRP  IL-6, composite complications  Bacteremia, IL-6, IL-1β, CRP, elastase, HLA-DR, ET  Bacteremia, IL-6, IL-1β, CRP, elastase, HLA-DR, ET    Bacteremia, IL-6, IL-1β, CRP, elastase, HLA-DR, ET  IL-6, IL-1β, TNF-α, IL-8, IL-10, +50 more mediators | Blood  Blood  Blood  Blood  Blood  Blood  Blood  Blood  Blood, P. fluid | Chinese herb medicine soup (KYL), 100 ml/day, oral, added to their routine treatment post abdominal surgery. Controls were treated by routine treatment only.  Polymyxin B immobilized fiber (PMX) was used to remove endotoxin by direct hemoperfusion (DHP).  Patients in the steroid group received 30 mg/kg of methylprednisolone 90 min before surgery, and in the control group received 50 ml physiologic saline intravenously.  After laparotomy, 25 patients were treated with continuous peritoneal lavage (CPL). The remaining 25 patients were drained without postoperative irrigation (Non-CPL).  Surgical necrosectomy included debridement through a 5-cm flank incision and postoperative lavage. Endoscopic transgastric necrosectomy was compared.  Open appendectomy using McBurney or inferior midline incision. Laparoscopic appendectomy using three trocar incision and 14 mm Hg CO2 Pneumoperitoneum.  Open surgical repair versus laparoscopic repair of perforated peptic ulcers.  Open cholecystectomy using a right subcostal incision; laparoscopic cholecystectomy using four trocar incisions and 14 mmHg CO2 pneumoperitoneum.  Patients were allocated to the  ABThera (125 mm Hg suction) versus Barker’s vacuum pack (20 mm Hg suction) in a 1:1 ratio after abbreviated laparotomy. | In both groups, CRP, IL-6, TNF-α levels peaked on POD1, then gradually declined. Patients treated with KYL declined faster. Also, patients in KYL group showed significant less SIRS/MODS. KYL appeared to inhibit release of inflammatory mediators and reduce post-operative SIRS.  Except for PAI-1 which decreased in the 16 surviving patients whereas increased in the 9 nonsurviving patients , other mediators did not change significantly after PMX-DHP treatment in either surviving or nonsurviving patients. PMX-DHP treatment had limited ability to remove inflammatory mediators.  IL-6 levels were lower in the steroid than in the control group on POD 1 (16.19 vs 91.46 pg/ml; P = 0.008) and on POD2 (13.71 vs 76.68 pg/ml; P = 0.002). IL-8, IL-10, CRP, and TNF-α also demonstrated similar trends to IL-6. The hospital stay in the steroid group was lower compared to the control group (10.5 versus 14.8 days; P < 0.05).  Comparing the inflammatory mediators in CPL with that of the non-CPL group, no significant difference could be established. Also no difference could be established for the clinical outcome (hospitalization, duration of intensive care and morbidity, p> 0.05).  IL-6 levels increased after surgical necrosectomy, whereas IL-6 levels decreased after endoscopy. The largest difference was seen at 24 h after intervention (P=.005). The composite clinical end point of death and major complications was also reduced in patients in the endoscopy group (20% vs 80%; RD, 0.60; P=0.03).  Open appendectomy caused a significant increase in neutrophil concentration, elastase, IL-1β, and IL-6. Bacteremia and endotoxemia were significantly higher in the open group compared with the laparoscopic group (P < 0.05).  Open surgical repair caused a significant increase in neutrophil concentration, neutrophil-elastase, IL-1 and IL-6, and decrease of human leukocyte antigen-DR (HLA-DR). Bacteremia and endotoxemia were significantly higher in the “open” group than in the laparoscopic group (p < .001).  Open cholecystectomy caused a quicker and greater increase of IL-1 and IL-6 than the laparoscopy did. Significant differences of IL-1, IL-6, CRP, HLA-DR, and endotoxemia between the groups were observed. Open cholecystectomy increased the incidence of bacteremia, endotoxemia and systemic inflammation compared with laparoscopy and caused lower transient immunological defense.  Median plasma concentrations of IL-6 at baseline were significantly higher among nonsurvivors (2263.3 pg/mL) than among survivors (679.0 pg/mL, P<0.001). Median plasma concentrations of IL-6 were significantly lower in the ABThera (637.4 pg/mL) versus Barker’s vacuum pack (2388.0 pg/mL) group at baseline (P = 0.03). There was no significant difference in clearance of the peritoneal fluid or plasma IL-6, -1β, -8, -10, or -12 p70 or TNF-α between 24 and 48 hours among patients between the groups. |

**Abbreviations**: CHDF, continuous hemodiafiltration; CRP, C-reactive protein; DHP, direct hemoperfusion; ENC, endotoxin neutralizing capacity; ET, endotoxin; HLA-DR, human leukocyte antigen-DR; ICAM-1, intercellular adhesion molecule 1; IL, interleukin; PAI-1, plasminogen activator inhibitor-1; P. fluid, peritoneal fluid; PMX, Polymyxin B immobilized fiber; TNF, tumor necrosis factor.
