# Supplementary material for: Inflammatory mediators in intra-abdominal sepsis or injury – a scoping review
Source: Crit Care. 2015 Oct 27;19:373. doi: 10.1186/s13054-015-1093-4 (PMC4623902; doi:10.1186/s13054-015-1093-4)
Supplement: Additional file 6: Table S6. — Preclinical mechanism. (DOCX 46 kb) [file 13054_2015_1093_MOESM6_ESM.docx]

Table S6. Summary of preclinical mechanistic studies of mediators in intra-abdominal sepsis/injury

| Study | Year | Type of model | No. of  animal | Mediators | Blood or P. fluid | Type of  mechanism | Outcomes and interpretation |
| --- | --- | --- | --- | --- | --- | --- | --- |
| Guo et al. [124]  Walley et al. [125]  Lane et al. [126]  Rongione et al. [127]  Wickel et al. [128]  Bathe et al. [129]  Lawlor et al. [130]  Martineau et al. [131]  Knoferl et al. [132]  Yao et al. [133]  Oda et al. [134]  Rezende-Neto et al. [135]  van Griensven et al. [136]  Ding et al. [137]  Ono et al. [138]  Rezende-Neto et al. [139]  Welborn et al. [140]  Deitch et al. [141]  Jarrar et al. [142]  Hildebrand et al. [143]  Frink et al. [144]  Kiank et al. [145]  Pimenta et al. [146]  Shimizu et al. [147]  Hsieh et al. [148]  Kan et al. [149]  Kan et al. [150]  Teke et al. [151]  Kiris et al. [152]  Liu et al. [153]  Hendriks et al. [154]  Shah et al. [155]  Wang et al. [156]  Zou et al. [157]  Kubiak et al. [158]  Fernandes et al. [159]  He et al. [160]  Jiang et al. [161]  Jung et al. [162]  Shah et al. [163]  Gui et al. [164]  Hirano et al. [165]  Hsu et al. [166]  Khailova et al. [167]  Zheng et al. [168]  Zou et al. [169]  Khailova et al. [170] | 1994  1996  1997  1997  1997  1998  1999  2000  2001  2001  2002  2002  2002  2003  2003  2003  2003  2004  2004  2005  2007  2007  2007  2007  2008  2008  2008  2008  2009  2009  2010  2010  2010  2010  2011  2012  2012  2012  2012  2012  2013  2013  2013  2013  2013  2013  2014 | Male rats - i.p. E. coli +/- rubber pieces  Mice - cecum ligation and puncture (CLP)  Female mice – SMA occlusion reperfusion (SMA I/R)  Female mice – CLP  Male mice – CLP  Porcine – SMA I/R injury  Male rats – hindlimb I/R injury  Male rats - abdominal E. Coli infusion  Male mice – 2 hits (shock + CLP)  Male rats - chemical peritonitis  Porcine – 2 hits (shock + ACS)  Male rats – ACS  Male mice – 2 hits (shock + CLP)  Male Rat – injury + endotoxemia  Male mice –  CLP + /-endotoxemia  Male rats – 2 hits (shock + ACS)  Knockout mice – I/R injury  Baboons – shock +/- thoracic duct ligation  Male rats - shock/resuscitation  Knockout mice – CLP  Male mice – shock resuscitation  Female mice – peritonitis  Male rats – Ischemia +/- reperfusion  Male rats – shock + resuscitation  Knockout mice – shock resuscitation  Rats – shock resuscitation  Male mice – shock resuscitation  Male rats – CLP, colon anastomosis  Rats – aortic ischemia/ reperfusion  Canine – portal vein occlusion/ reperfusion  Male rats – bacterial peritonitis  Swine – shock resuscitation + IAH  Male rats – CLP, colon anastomosis  Knockout mice – CLP  Swine – SMA ischemia/reperfusion + CLP  Knockout mice – CLP  Male rats – SMA I/R +/- lymph drain  Male rats – shock resuscitation  Mice – 2 hits: CLP +/- pneumonia  Swine – 2 hits: SMA I/R + CLP  Knockout mice – LPS peritonitis  Male rats – CLP  Male rats – shock resuscitation  Mice – CLP  Female mice – peritonitis  Knockout mice – CLP  Mice – CLP | 40 (10 per group)  110 (5 – 10 per group)  40 (10 per group)  56 (7 per group)  25 (5 per group)  14 (7 per group)  40 (3 groups)  85 (56 with E. coli infusion)  48 (8 per group)  58 (5 - 8 per groups)  20 (5 per group)  50 (10 per group)  50 (10 per group)  35 (8 - 9 per group)  80 (10 per groups)  60 (10 per group)  100 (9 – 12 per group)  15 (5 per group)  3 groups  40 (4 groups)  3 groups  137 (6 groups)  24 (8 per group)  36 (6 per group)  60 (10 groups)  36 (6 per group)  24 (6 per group)  48 (12 per group)  32 (8 per group)  18 (6 per group)  175 (15 – 20 per group)  6  40 (10 per group)  80 (9 groups)  5  40 (10 per group)  32 (8 per group)  18 (6 per group)  200 (10 groups)  16 (2 groups)  90 (9 groups)  117 (11 -19 per group)  30 (6 per group)  85 (12 groups)  64 (8 per group)  100 (10 – 50 per group)  36 (4 – 8 per group) | IL-6, TNF-α  IL-6, IL-10, TNF-α  IL-6, TNF-α, lung MPO  IL-6, TNF-α, IL-1β  WBC, ET,  liver AST, lung MPO and permeability  IL-6, TNF-α, ET  TNF-α, IL-1,  IL-1β, IL-2, IL-4, IL-6, TNF-α  PGE2, IL-6, TNF-α  Bacterial translocation  TNF-α, IL-1β, and IL-8, MPO  TNF-α, IL-6, IL-1β, lung MPO  TNF-α, lung neutrophil  IgA, bacterial translocation  TNF-α, IFN-γ, IL-10, IL-12, IL-18  CD11 on PMN, Lung elastase, permeability  IL-10, TNF-α, IL-1β, IL-6, lung MPO, liver AST  TNF-α, IL-6, sTNFR, elastase  IL-6, GST  IL-6, IL-10, TNF-α  TNF-α , IL-6, IL-10, MCP-1, KC, MIP-1  TNF, IL-6, IL-10, IL-12, IFN-γ, MCP-1  IL-6, CRP  IL-6, MPO, CINC-1, -3,  NF-κB, ICAM-1  GST, TNF-α, IL-6, IL-10, MCP-1,  MIP-1α  GST, MPO, HIFα, TNF-α, IL-6, ICAM-1, CINC-1, MIP-2  TNF-α, IL-6, KC, MCP-1, GST, MPO, iNOS, HIF-1α  TNF-α, IL-6, MPO, MDA, hydroxyproline (HP)  TNF-α, IL-1β, endothelium 1, MPO  TNF-α, IL-1β, MPO, ET  TNF-α, IL-6, IL-10  Neutrophil activation, IL-6, TNF-α  TNF-α, IL-6, protein C, MPO, MDA, HP, GST  TNF-α, IL-6, IL-10  TNF-α, IL-8, IL-6, IL-1β, IL-12, TGF-β, IL-10  IL-6, IL-10, TNF-a, IL-1b, KC, MCP-1, MIP-1  TNF-α, IL-1β, IL-6, ICAM-1, HMGB1, ET  TNF-α, IL-1β, IL-6  TNF-α, IL-6, G-CSF, IL-1β, IL-10  IL-6, TNF-α, CD62L, CD11b, CD18  IL-6, TNF-𝛼, HMGB1  TNF-a, IL-1β, IL-6, HMGB1  IL-1β, IL-6, TNF-α, CINC-1, CINC-3  TNF-α, IL-6  IL-6, TNF-α, IL-12p70, IL-10, IL-1β  IL-6, IL-10, TNF-α, KC, C3a, cfB  IL-6, TNF-α, MPO, COX-2 | Blood, MLN, liver  Blood, P. fluid, liver, lung  Blood, lung  Blood  Blood, lung  Blood (artery, hepatic or portal vein)  Blood (artery), liver  Blood, P. fluid  Blood, Kupffer cell  Blood, P. fluid, MLN, liver, lung, kidney  Blood (central or portal vein), BALF  Blood, lung  Blood, lung, liver, kidney  Blood, intestine  Blood, liver, MNCs  Blood, BALF, AST, creatinine  Blood, lung, liver  Blood, thoracic lymph  Blood, liver  Blood, lung, liver  Blood, Kupffer cell  Blood, P. fluid, lung, liver  Blood (cardiac, cava vein, portal vein)  Blood, liver  Blood, Kupffer cell  Blood, liver  Blood, liver, Kupffer cell  Blood, colon tissue  Blood, BALF  Blood (portal vein), lung  P. fluid  P. fluid  Blood, colon tissue  Blood, heart tissue  Blood, P. fluid  P. fluid, lung, liver  Blood, lymph, intestine tissues  Lung tissue  Blood, BAL, P. fluid  P. fluid  Blood  Blood  Lung tissue  Blood, colon tissue  Blood, P. fluid  Blood, P. fluid  Lung tissue | Rubber drain in abdominal sepsis  The sizes of CLP and inflammation; IL-10 and anti-IL-10 antibody and survival  IL-10 given before or after reperfusion injury and cytokine levels  IL-10 given before or after CLP sepsis and cytokine response  Anti-mouse neutrophil  antibody and remote organ injury  The release site of cytokines during or after SMA I/R injury  Cytokine release during I/R injury and anti-TNF antibody’s protection  Systemic and peritoneal cytokines with intra-abdominal infection  Selective COX-2 inhibitor and mediator release in I/R + CLP model  Bacterial translocation to blood and tissues  Inflammatory response to hemorrhage shock and abdominal hypertension  Inflammatory response to intra-abdominal pressure  Inflammatory response to one hit (shock) vs. two hits (shock + sepsis)  Gut permeability after trauma and endotoxemia  Inflammatory response and liver damage to CLP + LPS  Kinetics of maximal PMN priming by shock + ACS  IL-10 protection of lung and liver from I/R induced inflammation  Hemorrhage shock with thoracic duct ligation to lung, endothelium injury  Inflammation to hemorrhage shock plus ERK inhibitor  Inflammation in ICAM-1 knocked out mice with CLP sepsis  Inflammatory response in shocked mice treated with Finasteride  Survivor and inflammation in immunodefective mice with sepsis  The difference of IL-6 from the 3 sites after visceral ischemia and reperfusion  Inflammation and hepatic injury in rats treated with flutamide  Inflammation in MIP-1 knockout mice  Inflammation in rats treated with iNOS inhibitor  Inflammation and liver injury in mice treated with flutamide  Inflammatory response and tissue healing in rats following CLP  Lung injury to aortic I/R and treated with Tezosentan in rats  Inflammation to portal vein I/R and lymphatic ducts ligation  Peritoneal cytokine levels to mortality in rat peritonitis  Activation of neutrophils by P fluid after shock and ACS  Anti-inflammatory and antioxidant effects of HES 130/0.4 in CLP rats  Inflammation in TLR-2 knockout mice  Visceral ischemia/ abdominal sepsis and ACS  The role of transient receptor potential vanilloid 1 (TRPV1) in CLP sepsis  Lymph drainage to block the “gut-lymph” pathway, and tissue injury  HIF-1α inhibitor protects lung from shock induced injury  Inflammation and survival in mice with CLP sepsis plus pneumonia  Peritoneal fluid to activate neutrophils in I/R and septic swine  The protective role of CB2R to mice with LPS sepsis  The role of a RAS inhibitor in CLP septic rats  17b-Estradiol (E2) prevents shock induced lung injury  Mechanism of septic baby mice treated with oral lactobacilli  Mechanism of EH treating PGN-induced peritonitis  The role of TLR in septic mice with TLR knockout (MyD88 deletion)  Protection of oral lactobacilli from lung injury in baby mice | Bacterial translocation to MLN and the liver was observed in animals received either i.p. injection of 5 ml E.coli suspension or implantation of pieces of rubber in the abdomen, but was significantly increased in animals received both with a concomitant elevation of serum levels of TNF-α and IL-6.  Decreasing cecal-puncture diameter resulted in decreased mortality with delayed and decreased expression of TNFα and IL-6, and increased IL-10 levels. Lower IL-10 concentrations were associated with high TNFα and IL-6 concentrations in peritoneal fluid during severe sepsis. Administration of IL-10 or anti-IL-10 altered the balance of mediators and resulted in decreased or increased mortality, respectively.  Systemic levels of TNF-a, IL-6, and lung myeloperoxidase levels were substantially increased in animals undergoing superior mesenteric artery occlusion and reperfusion (SMA I/R) injury when compared to animals undergoing sham laparotomy alone. IL -10 reduced the severity of local and systemic inflammation mediators.  CLP produced a significant rise in serum TNF-α, IL-6, and IL-1β in untreated controls. Prophylactic or therapeutic administration of IL-10 significantly attenuated this early rise in serum cytokines. IL-10 was able to inhibit release of macrophage-derived mediators.  Serum endotoxin was 40-fold greater in the antibody-treated CLP animals than for non-CLP, and 4-fold greater than for saline CLP subjects, at the 6-hour time point (p< 0.05). The lung MPO and permeability were no difference between the normal control and those in the antibody treated groups.  TNF-α was produced in a partially perfused splanchnic bed during SMA clamping (e.g., pancreas, duodenum, liver, left colon). IL-6 was produced in gut during SMA clamping and was released when the SMA was unclamped. There was no apparent splanchnic release of endotoxin during or after SMA clamping in this model.  A significant peak in serum TNF-α (83.97 pg/mL) occurred at 30 min of reperfusion and returned to baseline by 60 min. No significant changes in TNF-α or IL-1 levels were measured during the ischemic period or at 6 h of reperfusion. I.v. injection of TNF-α antibody before reperfusion significantly diminished the number of dead hepatocytes.  Plasma cytokine concentrations were far below those in peritoneal fluid (p <0 .05). Plasma TNF-α concentrations increased up to 5-fold in septic rats compared with controls during the first 36 h (p < 0.05). Plasma IL-1β, IL-4 and IL-6 were not changed. Peritoneal TNF-α and IL-6 were 3 times greater in nonsurvivors than in survivors.  NS-398 markedly suppressed the elevation in plasma PGE2 and IL-6 levels following CLP. *In vitro* Kupffer cell IL-6 production after CLP was significantly reduced by *in vivo* NS-396 treatment. Activation of COX-2 following hemorrhage and sepsis up-regulates Kupffer cell IL-6 production.  Bacteria were cultured from the MLN and from the peritoneal fluid at 48 h post zymosan injection. No bacteria were cultured from the liver, lung or kidney. However, as the dose was increased, there was bacterial translocation to distal organs.  Portal and central vein cytokine levels were equivalent but were significantly higher in swine with shock and ACS than in other groups (sham, shock, or ACS alone). Sequential insults of ischemia reperfusion and ACS were associated with significantly increased portal and central venous cytokine levels and more severe lung injury than shock or ACS alone.  Intra-abdominal hypertension (20 mmHg for 60 or 90 min) caused a significant decrease in mean arterial pressure, increase in systemic levels of IL-6 and IL-1β. Lung neutrophil accumulation was significantly elevated only after abdominal decompression.  TNF-α levels significantly increased after 96 h (48 h after CLP, two hits). Thickened alveolar septa lesions were only seen in the lungs in the two hit group. Neutrophil adhesion to the pulmonary endothelium was significantly increased in the two hit group.  The damage of small intestinal mucosa in glutamine treated rats was remarkably alleviated. Addition with glutamine in total parental nutrition could reduce intestinal permeability and bacterial translocation caused by trauma and endotoxemia.  Serum IL-12 and IL-18 levels and liver MNCs, IL-12, IL-18, and IFN-γ production were significantly increased in CLP mice. Mice surviving from mild CLP peritonitis died of severe liver injury by subsequent injection of a sublethal dose of LPS, the mortality was reduced by anti-IL-12 and anti-IL-18 antibody treatment.  CD11b expression on PMN was the highest at 8 h post-shock/resuscitation. The ACS introduced 8 h post-shock provoked lung and liver injury, but did not if introduced at 2 or 18 h post-shock when there was no evidence of systemic PMN priming.  Exogenous IL-10 resulted in a decrease in the lung neutrophil infiltration in the IL-10−/−null mice. The endogenous IL-10 response to visceral ischemia-reperfusion attenuates the lung neutrophil infiltration and injury but has no effect upon either the hepatic injury or the systemic inflammatory response.  Lymph from the hemorrhage shock group collected during the early postshock period was cytotoxic for human endothelial cells (HUVECs) and increased HUVEC monolayer permeability almost 2-fold (P < 0.01). Diversion of thoracic duct lymph prevented shock/resuscitation induced lung injury.  The IL-6 and GST levels increased in vehicle-treated animals at 24 h after fluid resuscitation. Inhibition of intracellular signaling pathways by ERK inhibitor following hemorrhage/resuscitation resulted in nondetectable levels of IL-6 and decreased GST.  In C57B1/6 ICAM-1-/- rats undergoing CLP, the 96 h mortality rate was 12.5% with lower plasma concentrations of pro- and anti-inflammatory cytokines, compared to 45.5% in the WT group (p<0:05).  Plasma levels of TNF-α, IL-6, IL-10, MCP-1, KC, and MIP-1α increased after trauma-hemorrhage in vehicle-treated animals. Animals treated with finasteride before hemorrhage prevented this increase and Kupffer cell cytokine production after shock, which was abolished by treatment with the estrogen receptor antagonist.  Chronically stressed mice had increased lymphocyte apoptosis, ameliorated lethal shock responses but reduced the capacity to eradicate bacterial infection during mild sepsis.  After 2 h visceral and hind limb ischemia, serum IL-6 in samples collected at the heart cavity (223.6 pg/mL) was higher than portal (133.08 pg/ml) and posterior cava vein (127.58 pg/mL; p=0.01). Serum IL-6 at cava vein was similar to portal vein. No difference was recorded for CRP among groups and sites at 4 h after ischemia.  Flutamide attenuated hepatic injury, myeloperoxidase activity, NFκB activity, ICAM-1, IL-6, CINC-1 and CINC-3, restored the decreased plasma estradiol levels following hemorrhage shock. Coadministration of estrogen receptor antagonist prevented those salutary effects of flutamide.  A marked increase in serum GST, TNF-α, IL-6, IL-10, MCP-1, and MIP-1α and Kupffer cell cytokine production was observed in wild type mice, but not in the MIP-1α knockout mice, following shock.  Hemorrhage/resuscitation induced an increase in hepatic expression of iNOS, HIFα, ICAM-1, IL-6, TNF-α, and neutrophil chemoattractant protein levels, which was attenuated by administration of the iNOS inhibitor with less hepatic damage.  Flutamide administration reduced liver injury, with decreased levels of GST, myeloperoxidase activity, nitrotyrosine formation, lipid peroxidation, and cytokines/chemokines; rebalanced immune functions in male mice after trauma-hemorrhage.  Activated protein C treatment led to significant increases in anastomotic bursting pressures and tissue HP contents following CLP, along with decreases in MPO activity, plasma levels of TNF-α, and IL-6, (P<0.05).  Tezosentan significantly decreased aortic IR induced plasma level of TNF-α; lung tissue MDA and MPO; and protein concentration in bronchoalveolar lavage fluid. Tezosentan attenuated lung injury induced by aortic IR.  The levels of cytokines and endotoxin in the lymph from thoracic duct were significantly increased in portal vein occlusion group compared with control (P <0.05), which were significantly decreased after mesenteric lymphatic ducts ligation (P < 0.05).  Peritoneal cytokine levels were higher in nonsurvivors than in survivors (p <0.0001). At 24 h there were strong correlations between IL-6 and IL-10 (r= 0.93). A increased mortality was observed if IL-6, IL-10, or TNF-α levels exceeded 2, 1, or 0.2 ng/mL, respectively.  The levels of IL-6 and TNF-α were increased in peritoneal fluid at time of decompression and continued to be increased at 6 and 12 h thereafter. Peritoneal fluid significantly increased CD11b and CD18 expression on PMNs and monocytes.  HES130/0.4 treatment significantly inhibited CLP-induced increases in perianastomotic tissue MPO activity, MDA levels, NF-κB activation, and plasma levels of TNF-α, IL-6 and protein C. Colonic anastomotic bursting pressures, perianastomotic tissue HP contents, GSH levels were increased by HES 130/0.4 treatment.  TLR-2 knock-out mice show less cytokines levels in blood and tissue, less leukopenia in the peritoneum, and lower mortality than wild type littermates following CLP sepsis. TLR2-deficient mice had markedly improved cardiac function and survival during sepsis.  The lung, kidney, liver and intestine all demonstrated injury with progressive deterioration of organ function over the 48-hours following superior mesenteric artery I/R and abdominal sepsis, along with ACS development and increase in multiple cytokines in serum and peritoneal fluid. Three pigs died before 36 h.  TRPV1-knockout CLP mice exhibited significant hypothermia, hypotension, and organ dysfunction, along with decreased mononuclear cell integrity, increased cytokine levels, and decreased bacteria clearance when compared with CLP wild type mice.  The serum levels of HMGB1, endotoxin, and inflammatory factors in the SMA I/R group were higher than that in the I/R+ lymph drain group (P < 0.05). In the sham group there was no HMGB1 staining of the jejunum and ileum. In the I/R group, both the top of villi and the basement membrane were stained for HMGB1 in most areas.  Intratracheal delivery of HIF-1α inhibitor YC-1 ameliorated the accumulation of TNF-α, IL-1β, and IL-6 induced by shock/resuscitation; significantly attenuated lung injury, and reduced pulmonary HIF-1α and iNOS expression, pulmonary edema, and MPO activity.  CLP followed by MRSA pneumonia had a blunted systemic and local inflammatory response with diminished levels of IL-6 and IL-1β, and decreased local bacterial clearance, leading to increased mortality compared with either insult in isolation.  Significant increases in IL-6 (3.16 to 21.89 ng/mg, p=0.01 vs. baseline) and TNF-α (1.89 to 3.62 ng/mg) were noted in peritoneal fluid from septic swine. There was a significant increase in neutrophil activation as measured by cell surface marker expression of CD11b and CD18. Peritoneal fluid may serve as priming and activating stimulus for neutrophils.  Serum levels of TNF𝛼, IL-6, and HMGB1 were significantly higher and survival rate was lower in CB2R deficient mice as compared with the WT group after LPS administration. CB2R is a potential therapeutic target for the sepsis.  Treatment with a RAS inhibitor (PRRB) significantly suppressed serum concentrations of TNF-α, IL-1β, and HMGB1, but not IL-6, after CLP, with improved survival compared with control (P =0.023).  Estradiol (E2) prevents trauma-hemorrhage-induced lung damage, attenuated increase in lung myeloperoxidase activity, edema formation, inflammatory mediator levels, and apoptosis, which was blocked by co-administration of PI3K/Akt inhibitor (LY294002).  Lactobacilli administration at initiation of CLP can improve survival, intestinal epithelial homeostasis, and attenuation of the local and systemic inflammatory responses in septic pediatric mice.  Ephedrine hydrochloride (EH) increased IL-10 and decreased IL-6, TNF-α, IL-12 and IL-1β expression in macrophages treated with PGN. The anti-inflammatory role of EH was also demonstrated in mice of peritonitis induced by i.p. PGN injection.  Activation of TLR2, TLR3, and TLR4 markedly enhanced complement factor B (cfB) synthesis and release by macrophages. CLP sepsis increased cfB levels in the serum, peritoneum, and organs. MyD88 deletion attenuated cfB upregulation, with improved survival and cardiac function.  Lactobacilli treatment significantly attenuated lung injury in mice following CLP sepsis and reduced lung neutrophil infiltration, levels of IL-6, TNF-α, and gene expression of Cox-2, TLR-2, MyD88 and NFκB (p50/p105). |

**Abbreviations**: APC, activated protein C; BALF, bronchoalveolar lavage fluid; CINC, cytokine-induced neutrophil chemoattractant; CLP, cecum ligation and puncture; CRP, C-reactive protein; EH, ephedrine hydrochloride; ERK, extracellular signal–regulated kinase; ET, endotoxin; GST, glutathione S-transferase; HIF-α, hypoxia inducible factor-α; HMGB1, high mobility group box nuclear protein 1; HP, hydroxyproline; IAH/ACS, Intra-abdominal hypertension/ abdominal compartment syndrome; ICAM-1, intercellular adhesion molecule 1; IL, interleukin; iNOS, inducible nitric oxide synthase; i.p., intraperitoneal; i.v., intravenous; KC, keratinocyte-derived chemokine; MAD, malondialdehyde; MCP, monocyte chemoattractant protein; MIP, macrophage inflammatory protein; MLN, mesenteric lymph nodes; MPO, myeloperoxidase; NF-κB, nuclear factor kappa B; P. fluid, peritoneal fluid; SMA I/R, superior mesenteric artery ischemia and reperfusion; sTNF-R, soluble-TNF-receptors; TNF, tumor necrosis factor.
