# Supplementary material for: Inflammatory mediators in intra-abdominal sepsis or injury – a scoping review
Source: Crit Care. 2015 Oct 27;19:373. doi: 10.1186/s13054-015-1093-4 (PMC4623902; doi:10.1186/s13054-015-1093-4)
Supplement: Additional file 7: Table S7. — Preclinical intervention. (DOCX 36 kb) [file 13054_2015_1093_MOESM7_ESM.docx]

**Table S7**. Summary of preclinical intervention studies of mediators in intra-abdominal sepsis/injury

| Study | Year | Type of model | No. of  animal | Mediators | Blood or P. fluid | Type of  intervention | Outcomes and interpretation |
| --- | --- | --- | --- | --- | --- | --- | --- |
| Eskandari et al. [171]  Remick et al. [172]  Angele et al. [173]  Angele et al. [174]  Hogaboam et al. [175]  Villa et al. [176]  Balague et al. [177]  Jarrar et al. [178]  Oberbeck et al. [179]  Bauhofer et al. [180]  Pross et al. [181]  Kuhn et al. [182]  Steinberg et al. [183]  Bruhn et al. [184]  Suzuki et al. [185]  Yang et al. [186]  Özen et al. [187]  Karakoc et al. [188]  Chatzimavroudis et al. [189]  Kubiak et al. [190]  Yuan et al. [191]  Calisto et al. [192]  Herzig et al. [193]  Peng et al. [194]  Bangash et al. [195]  Chen et al.[196]  da Silva et al. [197]  Du et al. [198]  Fujimura et al. [199]  Teixeira-da-Cunha et al. [200]  Theobaldo et al. [201]  Matsuda et al. [202] | 1992  1995  1997  1998  1998  1998  1999  2000  2001  2002  2002  2005  2005  2006  2006  2006  2007  2008  2009  2010  2011  2012  2012  2012  2013  2013  2013  2013  2013  2013  2013  2014 | Mice – sepsis (CLP, LSP)  Mice – sepsis by CLP or LPS  Male mice – 2 hits: shock resuscitation + CLP (at 48 h)  Male mice – shock/ resuscitation + CLP  Female mice – CLP  Male mice – CLP  Mice – E coli peritonitis  Male rats – 2 hits: shock + CLP (at 20 h)  Mice – shock resuscitation +/- CLP  Male rats - stool peritonitis  Male rats – fecal peritonitis  Male rats – fecal peritonitis  Swine – SMA ischemia/ reperfusion + peritonitis  Male mice – CLP  Male rats – shock resuscitation +/- CLP (20 h)  Male mice – shock resuscitation  Rats – CLP + ileocolic anastomosis  Female rats – CLP  Rabbits – CLP with triple punctures  Swine – SMA clamping + fecal sepsis  Female mice – peritonitis  Rats – CLP  Mice – CLP  Male rats – CLP  Male rats – endotoxemia  Male mice – CLP  Male mice – CLP  Male rats – shock resuscitation  Male rats – aortic ischemia/reperfusion  Male mice – CLP  Male mice – CLP  Male mice – SMA clamping reperfusion | 15 groups  160 (8 groups)  60 (4 groups)  45 (3 groups)  100 (10 groups)  100 (12 – 18 per group)  360 (4 groups)  48 (16 per group)  85 (8 – 15 per group)  150 (7 groups)  80 (10 groups)  70 (10 per group)  12 (5-7 per group)  80 (10 per group)  80 (9 groups)  53 (3 groups)  32 (8 per group)  85 (5 – 6 per subgroup)  42 (6 per group)  12 (6 per group)  100 (10 – 35 per group)  77 (8 – 15 per group)  100 (8 groups)  58 (3 groups)  80 (10 groups)  48 (16 per group)  48 (8 per group)  180 (12 groups)  54 (6 groups)  80 (11 groups)  297 (5 – 10 per group)  50 (6 groups) | TNF-α, IL-6, ET  TNF activity  IL-6, IL-1, IL-2  IL-6, IL-1  MIP-2, IL-10, MCP-1  neutrophils, TNF-α, IL-6, IL-10  TNF-α, IL-6, IL-1  IL-6  TNF-α, IL-1β  TNF-α, IL-6, IL-10  IL-6, MPO, bacteremia  IL-6, IL-10  TNF-α, IL-1, IL-6, IL-8, IL-10, Elastase, MMP-2, MMP-9    MIP, IL-6, IL-10, TNF-α  IL-6, TNF, IL-2, IFN-γ  IL-6, IL-10, ALT, TNF, bacterial count  TNF-α, IL-6, MDA, GSH  TNF-α, IL-1β, IL-16  CRP, PCT, bacteremia, endotoxemia  TNF-α, IL-1β, IL-6, IL-8, IL-12, IL-10, C5a, CRP, TGF-β, PGE2, ET, IAP  TNF-α, IL-10, C3, bacterial count  TNF-α, IL-1β, IL-6  IL-6. MIP-2, bacterial count  IL-6, creatinine  TNF-α, IL-1β, IL-6 , IL-10 , Lactate, ALT, creatinine  TNF-α, IL-6, IL-10, IL-17, bacteria  TNF-a, IL-6, IL-10, MCP-1, bacteria  TNF-α, IL-6, IL-10  IL-1β, IL-6, IL-10, TNF-α, HMGB-1, elastase,  MCP-1/CCL2, nitric oxide, bacteria  TNF-α, IL -10, IL-6, ICAM-1, VCAM-1  TNF-α, IL-6 | Blood  Blood  Splenic macrophage  Splenocyte,  macrophage  P. fluid  Blood, BALF, P. fluid  Blood, P. fluid  Blood  Blood  Blood, P. fluid  Blood, P. fluid, lung tissue  Blood, P. fluid  Blood, BALF  Blood, P. fluid  Blood, BALF, Kupffer cells  Blood, ileum tissue, MLN  Blood, burst pressure  Blood, P. fluid  Blood  Blood, P. fluid, BALF  Blood, P. fluid  Blood  Blood, P. fluid  Blood  Blood, lung, heart  Blood, P. fluid  P. fluid  Blood, intestine  Blood  P. fluid  P. fluid, lung  Blood, lung, intestine | Anti-TNF serum was injected S.C. or i.p. before or at the time sepsis  Anti-TNF serum (i.v. 3 h before CLP, LPS)  25 mg/kg bwt flutamide SC at resuscitation as well as 24 and 48 h thereafter  A single dose (100 µg) of DHEA, or 100 µg/d of DHEA for 3 days, before CLP  L-NAME at 8 mg/kg i.p. at the time of CLP  Daily 100 mg/kg murine G-CSF S.C. 3 days before CLP and 3 days after CLP  Open abdomen or laparoscopic pneumoperitoneum for 30 min after E coli peritonitis  A tyrosine kinase inhibitor, AG 556 (7.5 mg/kg) i.p. at the middle of resuscitation  30 mg/kg DHEA 30 min before CLP s.c. A second injection of DHEA was at 24 h  No peritoneal lavage; saline lavage; or saline plus G-CSF20 g/kg S.C. 12 h before, 12 and 36 h after peritonitis.  Open peritoneal lavage versus laparoscopic lavage 1 h after fecal peritonitis.  Laparoscopic saline lavage versus lavage plus endotoxin inhibitor i.p. 2h after peritonitis  COL-3 (modified tetracycline, 200 mg/kg) added to the food 12 h before SMA clamping  Dexamethasone 0.1, 1, or 10 mg/kg i.p. after CLP  Androstenediol 1 mg/kg i.v. at the end of the resuscitation (20 h before CLP)  600 μg anti-HMGB1 antibody or 600 μg nonimmune rabbit IgG in 500 μL PBS, i.p.  Melatonin 5 mg/kg or 10 mg/kg i.v., daily for 5 days  The necrotic cecum was excised 6 h after CLP; or peritoneal lavage only  After 12 h of CLP, pneumoperitoneum or open abdomen for 60 – 180 min  TAC-NPT (-125 mmHg); or TAC passive drainage. Plus resuscitation, antibiotics, ventilation  1 mg human purified Complement 3 (HuC3), i.p. at 6 h post-colon ascendant stent  Oral diacerhein (100 mg/kg) 3 h after CLP and then once per day  Anti-CXCR3 IgG 100 μg i.v. 24 h pre-CLP, or 2 h or 6 h post-CLP  Ampicillin/sulbactam (125 mg/kg every 12 h) for 3 days  Dopexamine in infusion fluid for 4 h resuscitation at infusion rates of 0.5, 1, and 2 μg/kg/min  Resolvin D1 (100 ng) i.v. at the time of CLP  P. acnes-killed (0.4 mg/animal), 1, 2, 3, and 7 days before CLP  ST36 acupuncture point was punctured for 1.5 h immediately after hemorrhage.  Sivelestat solution (4 mL/kg/h)  1 mg/kg of rPAF-AH i.p.,15 minutes after the CLP  Hypertonic saline solution 7.5% (4 ml/kg) or normal saline 0.9% (34 ml/kg) was infused  10 mg/kg of FK866 (inhibitor of visfatin) i.p. at the beginning of reperfusion. | Anti-TNF antibody treatment of mice subjected to CLP significantly reduced TNF bioactivity but did not reduce mortality or pulmonary neutrophilic infiltration. Anti-TNF antibody treatment concomitant with LPS injection reduced plasma TNF activity, but did not reduce mortality.  Anti-TNF did not decrease pulmonary neutrophil sequestration, improve survival, or prevent the decrease in temperature. Inhibition of TNF fails to reduce mortality in clinically relevant models of sepsis.  At 4th post-CLP day, 23.1% of the vehicle-treated animals subjected to shock/resuscitation/sepsis were alive compared with 66.7% of the flutamide-treated animals. Flutamide not only improved the depressed immune functions but also the survival of animals after hemorrhage and subsequent sepsis.  DHEA treatment after hemorrhage-shock improved immune functions and survival from subsequent sepsis. Only 7 of 15 of the vehicle-treated animals were alive compared with 11 of 13 in the group receiving a single 100 µg dose of DHEA, and 12 of 13 in the group receiving DHEA 3 times.  Nitric oxide inhibitor (L-NAME) increased IL-10 and MCP-1 in peritoneal fluid after CLP, improved the survival (P = 0.033 vs. control).  A prophylactic regimen combining G-CSF and antibiotics might prevent infectious complications following intraabdominal surgery.  Bacterial numbers in peritoneal fluid and blood, and peritoneal IL-1 and IL-6 levels were significantly lower in the laparoscopic group than in the open group.  AG 556-treated rats showed significantly lower IL-6 (11.7 pg/mL) than in the vehicle group (281.5 pg/mL). Administration of AG 556 during resuscitation decreased the mortality rate to 25% (69% of the vehicle-treated rats) after CLP.  DHEA increased survival, accompanied by a reduction of TNF-α release, a restoration of the depressed DTH reaction, and an improved T-cellular immunity.  Cytokine levels in plasma were markedly lower than in the peritoneal fluid at all sampling times. The combination of lavage and G-CSF increased survival (P < 0.05), stimulated granulocyte phagocytic activity, and reduced the levels of IL-6 and TNF-α in peritoneal fluid.  Laparoscopic lavage with a CO2 pneumoperitoneum had no difference on the inflammatory reaction during the early postoperative phase, but with reduced neutrophil sequestration in lung tissue compared to open lavage.  Laparoscopic lavage plus endotoxin inhibitor i.p. caused a significant reduction of IL-6 in the peritoneal fluid, and increased survival compared to control.  COL-3 treatment prevented septic shock and ARDS, significantly decreased cytokine levels in plasma and BALF, reduced elastase activity and MMP-2 and MMP-9 activity in BALF.  Dexamethasone decreased plasma MIF at 18 h with 10 mg/kg dose only. It increased peritoneal IL-10 at 6 h.  Androstenediol markedly decreased plasma IL-6 and TNF-α levels, improved survival following shock and CLP by maintaining the immune cell functions.  Treatment with anti-HMGB1 antibody improved survival at 24 h and ameliorated ileal mucosal hyperpermeability, decreased bacterial translocation to MLNs, and with lower plasma IL-6 and IL-10.  The 10 mg/kg of melatonin had a more potent effect on the healing of colonic anastomosis than the 5 mg/kg dose in peritonitis. No significant changes for cytokines after melatonin treatment.  If the infectious source is present, lavage is not beneficial in terms of cytokines or survival. Peritoneal IL-6 level predicts death in intra-abdominal infections.  Serum procalcitonin was higher and survival was lower in groups with laparotomy. Bacteremia and endotoxemia were induced in all groups. White blood cell and C-reactive protein levels showed similar in all groups.  Systemic inflammation was significantly reduced in the NPT group and was associated with significant improvement in intestine, lung, kidney, and liver histopathology. NPT efficacy is partially due to an attenuation of peritoneal inflammation by the removal of ascites.  A single injection of HuC3 stabilized C3 levels for about 6 h, decreasing the 24 h mortality, reduced bacterial burden and attenuated organ injury in sepsis. No significant changes for cytokines.  Oral diacerhein improved survival during CLP sepsis, promoted downregulation of proinflammatory signaling cascades, attenuated increased levels of IL-1β, IL-6, and TNF-α, and reduced insulin resistance.  Treatment with anti-CXCR3 IgG significantly improved survival compared with nonspecific IgG plus Primaxin, along with reduced IL-6 and MIP-2 production.  Bactericidal antibiotics resulted in a transient acute kidney injury. Transient worsening of renal function may be a consequence of sepsis therapy.  Endotoxemia and laparotomy resulted in significant organ injury. Dopexamine at doses within the clinical range can attenuate TNF-α, IL-1β, IL-6 release, tissue leukocyte infiltration, and hence organ injury at doses that do not alter global hemodynamics or regional microvascular flow.  Resolvin D1 treatment improved survival, enhanced bacterial clearance, suppressed neutrophils in peritoneal lavage fluid, reduced the blood cytokines, and decreased the apoptosis rate of CD3+T lymphocytes of the thymus.  Prophylactic treatment with P.acnes-killed increased the survival of the animals, reduced the number of bacteria in the peritoneal cavity with increased migration of leukocytes.  Acupuncture at ST36 attenuates the systemic inflammatory response, protects intestinal barrier integrity, improves organ function and survival rate after hemorrhagic shock via activating the cholinergic anti-inflammatory mechanism.  Treatment with sivelestat significantly improved survival rate, lung permeability and edema, and decreased levels of creatinine, IL-6, IL-10, and neutrophil elastase activity.  Exogenous rPAF-AH improved bacterial clearance, decreased the levels of KC, IL-6, TNF and MIF while it increased the levels of IL-10 and MCP-1/CCL2.  Neutrophil infiltration, ICAM-1, and CXCL-1 in lung were reduced by hypertonic saline compared to normal saline. Neutrophil in peritoneal lavage was increased in 24 h, with improved animal survival.  FK866 treatment significantly attenuated intestinal and lung injury by inhibiting cytokine production, and NF-κB activation. |

**Abbreviations**: ABG, arterial blood gas; BALF, bronchoalveolar lavage fluid; CLP, cecum ligation and puncture; CXCL-1, neutrophil chemoattractant chemokine motif ligand 1; CRP, C-reactive protein; DHEA, dehydroepiandrosterone; GST, glutathione S-transferase; HMGB1, high mobility group box nuclear protein 1; IAP, intra-abdominal pressure; ICAM-1, intercellular adhesion molecule 1; IL, interleukin; i.p., intraperitoneal; i.v., intravenous; KC, keratinocyte-derived chemokine; LPS, lipopolysaccharide; MAD, malondialdehyde; MAP, mean arterial pressure; MCP, monocyte chemoattractant protein; MIP, macrophage inflammatory protein; MLN, mesenteric lymph nodes; MPO, myeloperoxidase; NF-κB, nuclear factor kappa B; NPT, negative pressure therapy; rPAF-AH, recombinant platelet-activating factor-acetylhydrolase; PAP, pulmonary arterial pressure; PCT, procalcitonin; P. fluid, peritoneal fluid; SC, subcutaneous; SMA, superior mesenteric artery; TAC, temporary abdominal closure; TNF, tumor necrosis factor; VCAM, vascular cell adhesion molecule.
